# The Interplay of Cofactor Interactions and Post-translational Modifications in the Regulation of the AAA+ ATPase p97

**DOI:** 10.3389/fmolb.2017.00021

**Published:** 2017-04-13

**Authors:** Petra Hänzelmann, Hermann Schindelin

**Affiliations:** Rudolf Virchow Center for Experimental Biomedicine, University of WürzburgWürzburg, Germany

**Keywords:** p97, AAA+ ATPase, conformational changes, protein quality control, protein disassembly, cofactor diversity, post-translational modification

## Abstract

The hexameric type II AAA ATPase (ATPase associated with various activities) p97 (also referred to as VCP, Cdc48, and Ter94) is critically involved in a variety of cellular activities including pathways such as DNA replication and repair which both involve chromatin remodeling, and is a key player in various protein quality control pathways mediated by the ubiquitin proteasome system as well as autophagy. Correspondingly, p97 has been linked to various pathophysiological states including cancer, neurodegeneration, and premature aging. p97 encompasses an N-terminal domain, two highly conserved ATPase domains and an unstructured C-terminal tail. This enzyme hydrolyzes ATP and utilizes the resulting energy to extract or disassemble protein targets modified with ubiquitin from stable protein assemblies, chromatin and membranes. p97 participates in highly diverse cellular processes and hence its activity is tightly controlled. This is achieved by multiple regulatory cofactors, which either associate with the N-terminal domain or interact with the extreme C-terminus via distinct binding elements and target p97 to specific cellular pathways, sometimes requiring the simultaneous association with more than one cofactor. Most cofactors are recruited to p97 through conserved binding motifs/domains and assist in substrate recognition or processing by providing additional molecular properties. A tight control of p97 cofactor specificity and diversity as well as the assembly of higher-order p97-cofactor complexes is accomplished by various regulatory mechanisms, which include bipartite binding, binding site competition, changes in oligomeric assemblies, and nucleotide-induced conformational changes. Furthermore, post-translational modifications (PTMs) like acetylation, palmitoylation, phosphorylation, SUMOylation, and ubiquitylation of p97 have been reported which further modulate its diverse molecular activities. In this review, we will describe the molecular basis of p97-cofactor specificity/diversity and will discuss how PTMs can modulate p97-cofactor interactions and affect the physiological and patho-physiological functions of p97.

## Introduction

**Figure 1:**
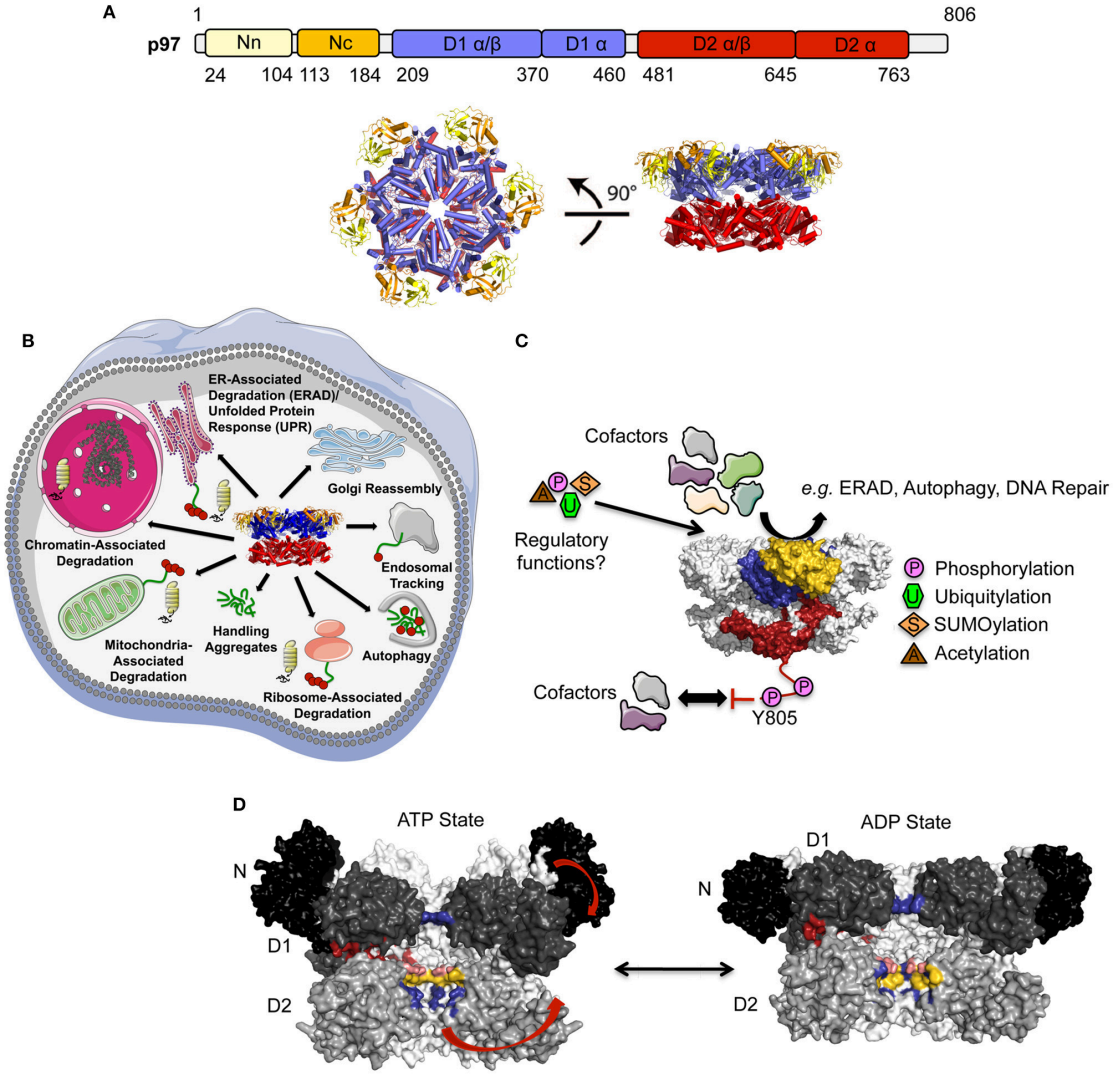
**Structure and function of p97. (A)** Top, Domain architecture of p97. The N domain can be further subdivided into an N- (Nn, colored in yellow) and a C-terminal (Nc, colored in orange) subdomain, the two ATPase domains (colored in blue and red, respectively) each feature a larger α/β and a smaller α-helical subdomain. Bottom, top (left) and side views (right) of the p97 hexamer (pdb entry 3CF1; Davies et al., 2008) (adapted from Buchberger et al., 2015). **(B)** Cellular functions of p97. **(C)** Regulation of p97 function by cofactor interactions and post-translational modifications (PTMs). p97 is colored as in **(A)** for one subunit and gray for the remaining subunits. **(D)** Conformational changes of p97 upon ATP binding/hydrolysis. Molecular surface of the p97 central cavity (side view) in the ATP- and ADP-bound states (pdb entries 5FTN and 5FTK; Banerjee et al., 2016) with two monomers in black/dark/medium (N/D1/D2) gray and two monomers in light gray. For clarity, the two monomers in the front are not shown. The restriction in the D1 domain (His-gate) is shown in blue. Catalytically important positively charged (Arg586 and Arg599, colored in blue), negatively charged (Glu544, colored in pink), and hydrophobic (Trp551 and Phe552, colored in yellow) residues lining the D2 pore are indicated. Conformational changes are indicated with arrows and the opening at the D1D2 interface is shown in red.

p97 (also known as VCP, Cdc48, and Ter94) belongs to the functionally highly diverse AAA+ (ATPase associated with various cellular activities) superfamily of proteins, which is characterized by conserved ATPase core domains. Through specific structural elements, like for example additional domains and insertions as well as different oligomeric arrangements, they act as molecular motors by using conformational changes induced by ATP hydrolysis to perform mechanical work on many different substrates (reviewed in Erzberger and Berger, 2006; Wendler et al., 2012). p97 is a type II AAA+ protein composed of two hexameric ATPase rings (formed by its D1 and D2 domains) that stack on top of each other and an additional N-terminal domain important for cofactor and substrate binding (DeLaBarre and Brunger, 2003, 2005; Davies et al., 2008) (Figure 1A). Like all hexameric AAA+ ATPases p97 features a central cavity or pore lined by putative substrate interacting loops. Further members belonging to this group, referred to as NSF/Cdc48/Pex family, are the N-ethylmaleimide-sensitive fusion protein (NSF) involved in vesicular transport processes (reviewed in Zhao and Brunger, 2016), SPATA5 (spermatogenesis associated 5) and NVL (nuclear VCP-like) (Drg1 and Rix7 in yeast) involved in ribosome biogenesis (reviewed in Kressler et al., 2012) as well as PEX1 and PEX6 involved in peroxisome biogenesis (reviewed in Grimm et al., 2016). A feature unique to p97 is its 76 amino acid long, unstructured C-terminal extension, which is highly flexible and is involved in the regulation of the ATPase activity and cofactor assembly, the latter being modulated by phosphorylation (Li et al., 2008; Ewens et al., 2010; Niwa et al., 2012).

p97 participates in many different cellular pathways involved in the regulation of protein homeostasis, membrane fusion and vesicular trafficking as well as chromatin-associated functions (reviewed in Meyer et al., 2012; Meyer and Weihl, 2014). In all these processes p97 extracts or disassembles ubquitylated substrates from membranes, chromatin or, in general, from large protein complexes often, but not always, resulting in downstream degradation by the proteasome (Figure 1B): (i) p97 has been shown to extract different ubiquitylated proteins from chromatin in processes such as cell cycle regulation, transcriptional and replication stress responses, several DNA repair processes (nucleotide excision repair, double strand break repair), or replication. Subsequently, these proteins are either degraded by the proteasome or recycled to modulate the dynamics of chromatin regulators (reviewed in Franz et al., 2016); (ii) p97 is also involved in various membrane trafficking processes, including Golgi reassembly at the end of mitosis and in endocytosis (reviewed in Meyer, 2005; Bug and Meyer, 2012); (iii) p97 is a key player in multiple protein quality control pathways mediated by the ubiquitin proteasome system and autophagy. It is involved in the extraction of misfolded proteins from the ER (ER-associated degradation, ERAD; reviewed in Stolz et al., 2011; Wolf and Stolz, 2012) and similarly translocates damaged mitochondrial proteins into the cytosol in a process called outer mitochondrial membrane associated degradation (OMMAD; Heo et al., 2010; Xu et al., 2011; Hemion et al., 2014); p97 is also part of the ribosome-quality control complex (RQC), which is involved in the degradation of stalled nascent peptides (ribosome-associated degradation; Brandman et al., 2012). Recently, it could be shown that p97 is involved in the removal of damaged lysosomes by autophagy (Papadopoulos et al., 2017). Due to its participation in essential cellular processes, p97 has been linked to pathophysiological states including cancer, neurodegenerative disorders and premature aging (reviewed in Chapman et al., 2011; Fessart et al., 2013; Franz et al., 2014; Tang and Xia, 2016). Mutations of p97 are causative of three protein aggregation diseases (proteinopathies; reviewed in Tang and Xia, 2016): Multisystem Proteinopathy (MSP), Familial Amyotrophic Lateral Sclerosis (FALS) and Charcot-Marie-Tooth Disease, Type 2Y (CMT2Y).

The functional diversity of p97 is regulated, amongst other mechanisms, by a large number of regulatory cofactors, which either associate with the N-terminal domain or interact with the extreme C-terminus via distinct binding motifs/domains and target p97 to specific cellular pathways, sometimes requiring the simultaneous association with more than one cofactor (reviewed in Buchberger et al., 2015) (Figure 1C). Furthermore, post-translational modifications (PTMs) like SUMOylation, ubiquitylation, palmitoylation, acetylation, and phosphorylation of p97 have been identified by site-specific techniques and/or high throughput proteomics (Fang et al., 2016; PhosphoSitePlus, http://www.phosphosite.org, Hornbeck et al., 2015). More importantly, these modifications were proposed to modulate the diverse molecular activities of p97.

p97 contains 12 ATP binding sites, 6 in each ATPase ring, which are located at the interface of adjacent monomers. Upon ATP-binding and hydrolysis significant conformational changes occur, which are transmitted via long flexible linkers from the D2 domain to the D1 domain and further to the N domain (Figure 1D)(Banerjee et al., 2016; Na and Song, 2016; Schuller et al., 2016; reviewed in Xia et al., 2016). These conformational changes, which are regulated by intradomain (within the same protomer; Ye et al., 2003; Chou et al., 2014) and interdomain (between adjacent protomers; Huang et al., 2012; Li et al., 2012; Hänzelmann and Schindelin, 2016b) signaling mechanisms include: (i) Opening and closing of the D2 pore; (ii) Rotational movement of the ATPase rings; (ii) Up and down movements of the N domain. In addition, through these conformational changes the D1 and D2 domains move slightly apart from each other forming an additional channel leading into the D2 pore (Na and Song, 2016). The upper part of the D2 pore has a constriction near the center, which is formed by the six side chains of the D1 residue His317, a structural element referred to as the His-gate (DeLaBarre and Brunger, 2003, 2005; Hänzelmann and Schindelin, 2016b). There are two layers of pore loops lining the D2 pore, a smaller one composed of aromatic amino acids and a longer one featuring both negatively and positively charged residues. An intersubunit signaling network (ISS) has been identified that couples the conformation of the putative substrate-translocating pore to the nucleotide state of the cis-subunit, which is then transmitted to the trans-subunit and coordinates in this way ATP hydrolysis in adjacent monomers (Hänzelmann and Schindelin, 2016b). In addition, the ISS is involved in signal transmission from the D2 domain via the D1D2 linker to the D1 domain of the adjacent monomer, a mechanism called interprotomer motion transmission (Huang et al., 2012; Li et al., 2012).

In this review, we will focus on the molecular basis of p97-cofactor specificity/diversity and will discuss how PTMs can modulate p97-cofactor interactions and affect the physiological and patho-physiological functions of p97.

## Molecular insights into p97 cofactor diversity

The participation of p97 in highly diverse cellular processes is regulated by the association with a large number of cofactors (reviewed in Yeung et al., 2008; Stolz et al., 2011; Meyer et al., 2012; Buchberger et al., 2015). Known cofactors are typically multi domain proteins composed of specific p97 binding modules and additional domains which, for example, function in the recognition of ubiquitylated target proteins, possess catalytic domains for substrate processing or transmembrane domains amongst others. So far about 30 cofactors have been identified with the latest entries to the list being published in 2016 (Arumughan et al., 2016), hence the number is expected to further increase. Based on their function, cofactors can be divided into three major classes: (i) Substrate-recruiting cofactors like UBA-UBX proteins and UFD1-NPL4: these cofactors link substrates to p97 and contain, beside a p97 binding motif/domain, additional ubiquitin binding domains/motifs, which target ubiquitylated substrates; (ii) Substrate processing cofactors like ubiquitin (E3) ligases, deubiquitinases (DUBs) and peptide N-glycanase (PNGase,), which process ubiquitylated, and N-glycosylated substrates; (iii) Regulatory cofactors like the UBX proteins UBXD4 and ASPL (also known as TUG and UBXD9) as well as SVIP, which may sequester or recycle p97 hexamers. Despite the large number of cofactors, they interact via a small number of conserved binding modules (reviewed in Buchberger et al., 2015). Although a few cofactors bind via their PUB (PNGase/UBA or UBX containing proteins) or PUL (PLAP, Ufd3p, and Lub1p) domain to the unstructured C-terminal tail of p97, the majority of cofactors interact with the N-terminal domain either via a UBX (ubiquitin regulatory X)/UBXL (UBX-like) domain or three linear binding motifs, called VCP-interacting motif (VIM), VBM (VCP-binding motif), and SHP (BS1, binding segment). Molecular insights have been obtained for all interacting domains/motifs from corresponding p97-ligand complex structures as reviewed below.

### UBX and UBXL domains

**Figure 2:**
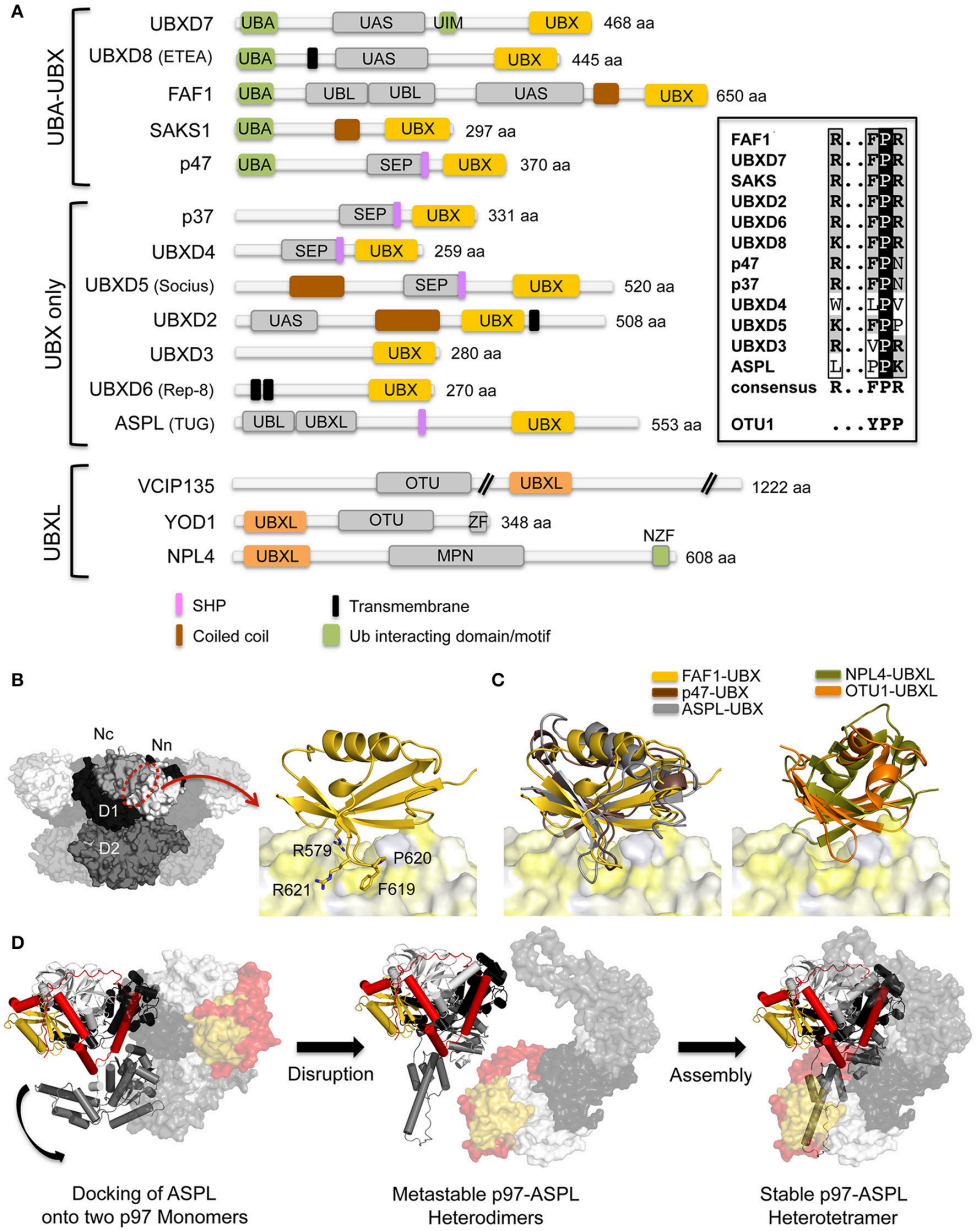
**Interaction of p97 with cofactors containing UBX and UBXL domains. (A)** Domain architecture of mammalian UBX and UBXL domain cofactors. A sequence alignment of the R…FPR motif in the UBX domains is shown on the right. MPN, (Mpr1, Pad1 N-terminal); NZF, NPL4 zinc finger; OTU, ovarian tumor; SEP, Shp, eyes-closed, p47; SHP, binding segment 1 (BS1); UAS, domain of unknown function found in FAF1 and other proteins; UBA, ubiquitin-associated; UBL, ubiquitin-like; UBX, ubiquitin regulatory X; UBXL, UBX-like; UIM, ubiquitin-interacting motif; ZF, zinc finger. **(B)** Left, molecular surface of p97 [N domain in white (Nn)/light gray (Nc), D1 in black, D2 in dark gray] with the UBX/UBXL binding site indicated. Right, FAF1-UBX—p97 N complex (pdb entry 3QQ8; Hänzelmann et al., 2011). The UBX domain (colored in gold) is shown in cartoon representation and p97 N as molecular surface (colored in shades of yellow according to hydrophobicity). The R…FPR motif is shown in stick representation. **(C)** Left, superposition of the UBX domains from FAF1 (pdb entry 3QQ8, colored in gold; Hänzelmann et al., 2011), p47 (pdb entry 1S3S, colored in brown; Dreveny et al., 2004) and ASPL (pdb entry 5IFW, colored in gray; Arumughan et al., 2016) bound to p97 N colored as in **(B)**. Right, superposition of the UBXL domains of NPL4 (pdb entry 4RV0, colored in olive; Hao et al., 2015) and OTU1 (pdb entry 4KDI, colored in orange; Kim et al., 2014) bound to p97 N colored as in **(B)**. **(D)** Disassembly of p97 hexamers through the interaction with the ASPL-UBX domain and formation of the stable p97-ASPL heterotetramer via metastable p97-ASPL heterodimers. For clarity, only two monomers of p97 are shown. One heterodimer is shown in cartoon representation and the other in surface representation. The ASPL-UBX domain is colored in yellow with the N- and C-terminal extensions in red and p97 in light gray (N domain), dark gray (D1 domain) and gray (D2 domain) (pdb entry 5IFW; Arumughan et al., 2016). The curved arrow indicates the reorientation of the D2 domain.

UBX and UBXL domains both consist of a ubiquitin-like fold. UBX proteins can be sub-divided into two families (Figure 2A): (i) UBA-UBX proteins, which also contain a UBA (ubiquitin-associated) domain that can bind to ubiquitylated substrates; (ii) UBX-only proteins. Molecular insights into the p97-UBX domain interaction have been revealed by crystal structures of the N domain in complex with the FAF1-UBX (Hänzelmann et al., 2011; Kim et al., 2011a; Lee et al., 2013) and the UBXD7-UBX (Li et al., 2017), p97-ND1 in complex with the p47-UBX (Dreveny et al., 2004) and full-length p97 in complex with the ASPL-UBX domain (Arumughan et al., 2016) (Figures 2B,C). A common feature is that the UBX domain interacts with the N domain via a conserved R…FPR signature motif (Figures 2A,B) located in a loop connecting two β-strands, which inserts into a hydrophobic binding pocket located in between the two subdomains of the N domain. The FPR motif adopts a cis-proline configuration, a rarely observed cis-Pro touch-turn structure, also called a FcisP touch-turn motif (Kang and Yang, 2011). In contrast to the UBX domains of FAF1, UBXD7 and p47, the N- and C-terminal regions of the UBX domain in ASPL contain unique structural extensions with the C-terminal extension in extensive contact with the β-grasp fold of the UBX domain (Figure 2D). Biochemical and structural data have shown that, as in other UBX proteins, the conserved cis-Pro touch-turn motif is important for the initial association with p97 hexamers. Subsequently an α-helical lariat structure formed by the N-terminal extension dissociates the p97 hexamer into monomers, resulting in the formation of a metastable p97-ASPL heterodimer (Arumughan et al., 2016). The α-helical lariat in ASPL is a flexible structure that directly targets the D1:D1 interprotomer interface in p97 hexamers, a region crucial for oligomer stability (Figure 2D). The p97-ASPL heterodimers subsequently oligomerize into (p97-ASPL)_2_ heterotetramers, accompanied by a reorientation of the D2 ATPase domain, leading to the inhibition of its ATPase activity.

In analogy to the FPR loop of UBX proteins, the crystal structure of the UBXL domain of the DUB OTU1 (yeast homolog of mammalian YOD1) in complex with the N domain also features a loop (YPP motif) inserting into the hydrophobic pocket (Kim et al., 2011a), whereas the UBXL domain of NPL4 does not feature an extended loop and binds differently (Isaacson et al., 2007; Hao et al., 2015) (Figure 2C). Despite displaying only a low degree of sequence identity, UBXL and UBX domains adopt a similar structure and bind in a similar position with respect to the N domain, yet the interaction modes and relative positions are specific for each protein.

### VIM- and VBM binding motifs

**Figure 3:**
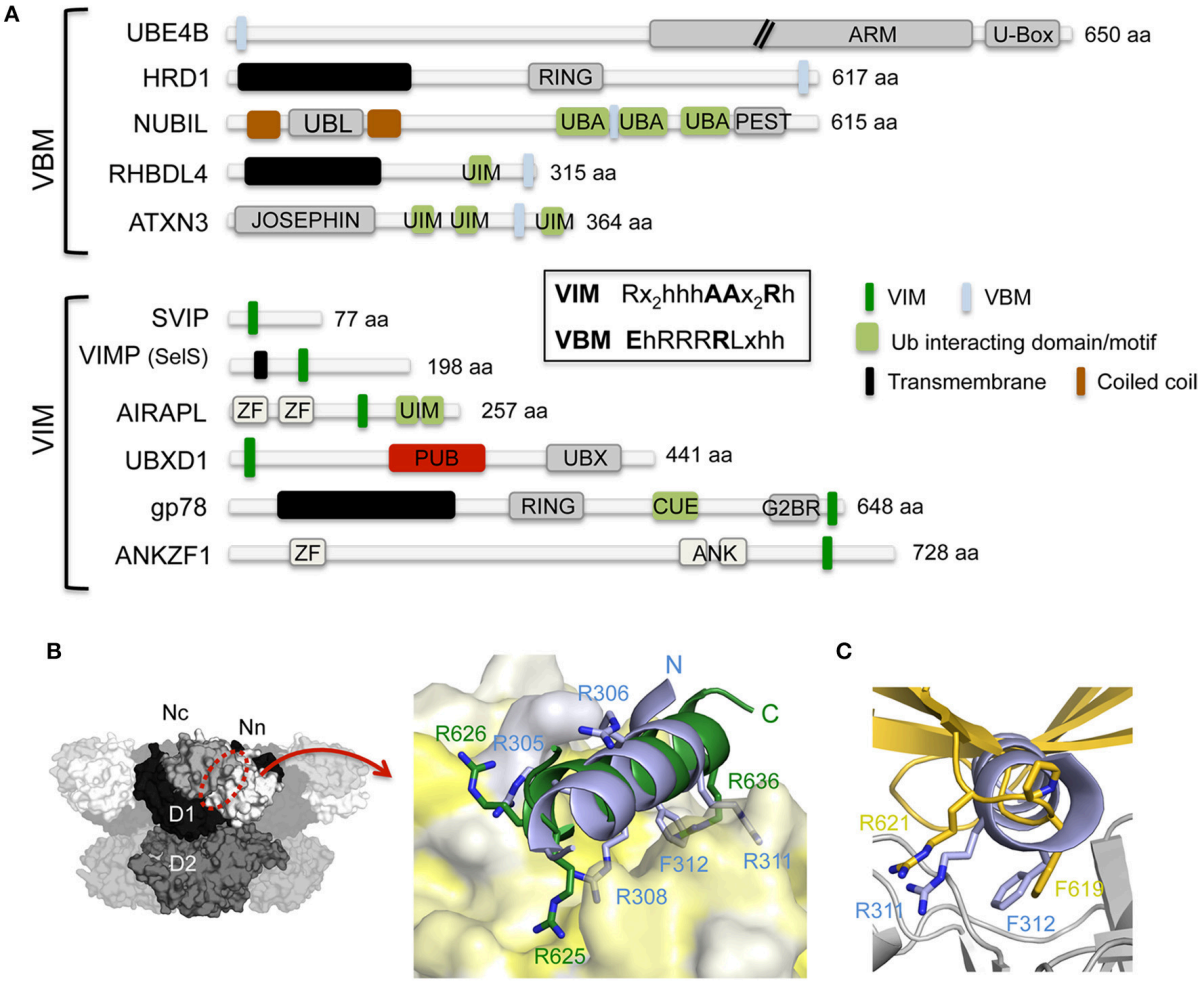
**Interaction of p97 with cofactors harboring either a VBM or VIM binding motif. (A)** Domain architecture of mammalian cofactors with a VBM or VIM binding motif. The consensus sequences for both binding motifs are shown in the inset. ANK, ankyrin repeat; ARM, armadillo/beta-catenin-like repeats; CUE, coupling of ubiquitin conjugation to endoplasmic reticulum degradation; G2BR, Ube2g2-binding region; JOSEPHIN, deubiquitinase domain; PEST, PEST motif, Pro, Glu, Ser and Thr rich sequence; PUB, PNGase/UBA or UBX containing proteins; RING, RING (Really Interesting New Gene) finger; U-Box, UFD2-homology domain; UBA, ubiquitin-associated; UBL, ubiquitin-like; UIM, ubiquitin-interacting motif; VBM, VCP-binding motif; VIM, VCP-interacting motif; ZF, zinc finger. **(B)** Left, molecular surface of p97 [N domain in white (Nn)/light gray (Nc), D1 in black, D2 in dark gray] with the VIM/VBM binding site indicated. Right, cartoon representation of a superposition of the gp78 VIM (pdb entry 3TIW, colored in green; Hänzelmann and Schindelin, 2011) and RHBDL4 VBM (pdb entry 5EPP, colored in light blue; Lim et al., 2016a) binding motifs in complex with the p97 N domain (molecular surface colored according to hydrophobicity). Key interactions are shown in stick representation. **(C)** Cartoon representation of a superposition of the RHBDL4 VBM (pdb entry 5EPP, colored in light blue; Lim et al., 2016a) and the FAF1-UBX (pdb entry 3QQ8, colored in gold; Hänzelmann et al., 2011) p97 N (colored in light gray) complexes. The side chains of the RF dipeptide of the VBM and the FPR motif of the VIM are shown in stick representation.

The VIM and VBM, which have been identified in several unrelated proteins (Figure 3A), are both linear polypeptide stretches enriched in positively charged amino acids that adopt an α-helical conformation. The crystal structure of the N domain in complex with a peptide covering the VIM binding motif of gp78 revealed that the α-helical motif interacts with the hydrophobic binding pocket located in between the two subdomains on the N domain (Hänzelmann and Schindelin, 2011) (Figure 3B). The VIM of gp78 contains, beside the two conserved arginines of the signature motif, a third non-conserved arginine (underlined in the consensus sequence) in front of the first arginine (RRx_5_AAx_2_Rh). All three arginines are important, with the highly conserved last arginine (Arg636 in gp78) being pivotal, and they engage in several electrostatic interactions as well as hydrophobic interactions via the aliphatic part of their side chains with the N domain. The crystal structure of the N domain in complex with the VBM of RHBDL4, a protein crucial for the retro-translocation of polyubiquitylated substrates in the ERAD pathway (Fleig et al., 2012), revealed a highly analogous overall spatial arrangement of the VBM and VIM with respect to the N domain (Lim et al., 2016a) (Figure 3B). Interestingly, the directionality of the α-helices is opposite in both structures. Highly conserved basic residues in VBMs (consensus EhRRRRLxhh; h, hydrophobic residue; x, any amino acid; hh = RF in RHBDL4) and VIMs (consensus Rx_2_h_3_AAx_2_Rh; h, hydrophobic residue; x, any amino acid) are important to maintain the N domain interaction by contributing the majority of the ionic and hydrogen bonded interactions in the interface. However, the RHBDL4 VBM-N domain structure revealed a novel binding mode, which unexpectedly combined the two types of p97-cofactor specificities observed in the UBX and VIM interactions. Specifically, the RF motif in RHBDL4 VBM corresponds to the FPR motif in UBX (Figure 3C), and the RRR motif in VBM (RRhRLxRF) corresponds to the RRR motif in the gp78 VIM (RRx_2_h_3_AAx_2_Rh) (Figure 3B).

The binding pocket formed in the Nn and Nc lobes provides a sterically unopposed interface for the interaction of the various p97 cofactor proteins. Proteomic studies identified phosphorylation, ubiquitylation, and mono-methylation sites in the VIM/VBM binding motifs of different proteins (Hornbeck et al., 2015), thus indicating that PTMs control the interaction with p97.

### SHP-binding motif

**Figure 4:**
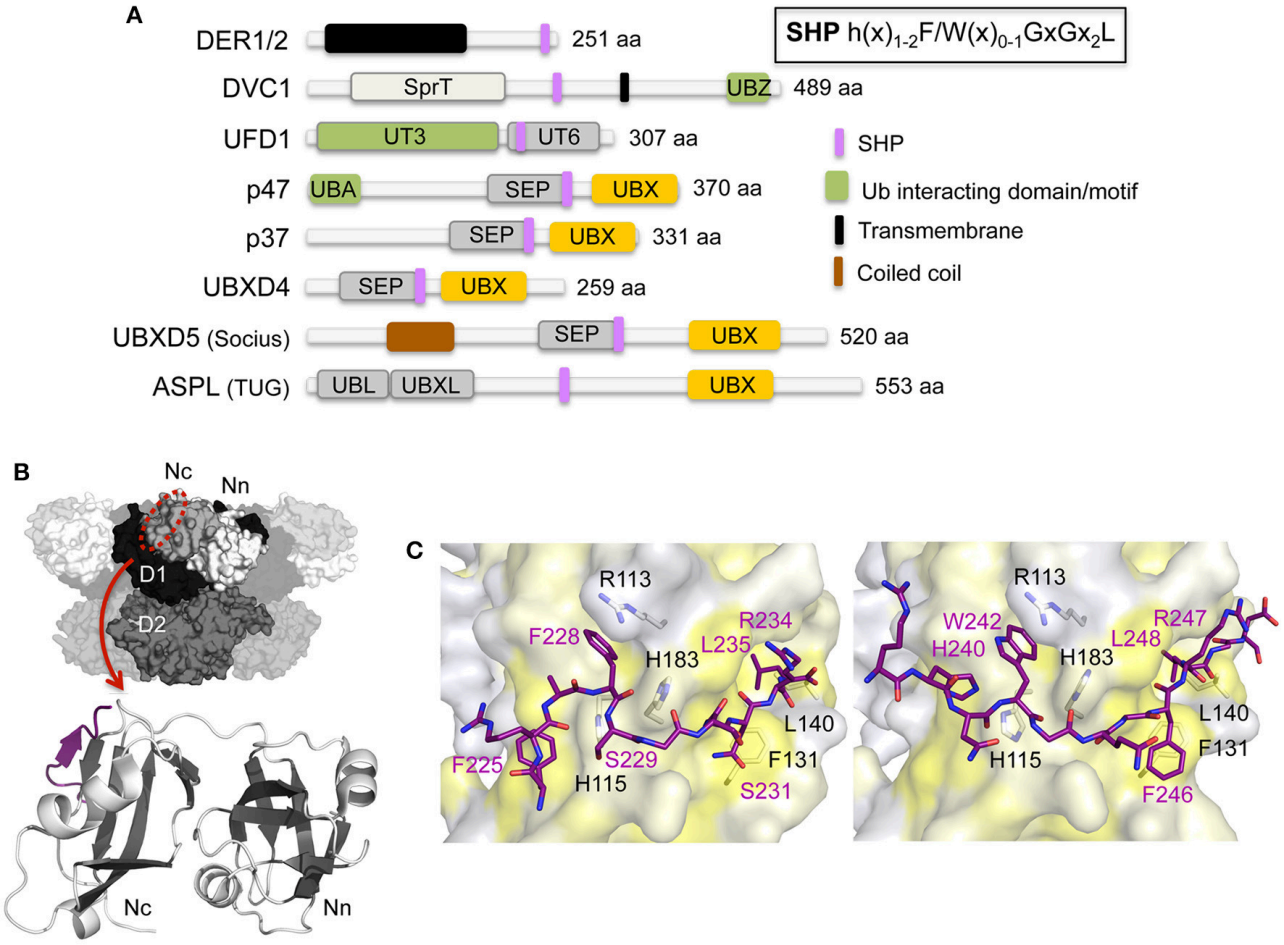
**Interaction of p97 with cofactors harboring a SHP binding motif. (A)** Domain architecture of mammalian cofactors with a SHP binding motif. The consensus sequence for the SHP binding motif is shown in the inset. SEP, Shp, eyes-closed, p47; SHP, binding segment 1 (BS1); SprT, SprT-like; UT3 and UT6, UFD1 domains; UBA, ubiquitin-associated; UBL, ubiquitin-like; UBX, ubiquitin regulatory X; UBXL, UBX-like; UBZ, ubiquitin-binding zinc finger; UIM, ubiquitin-interacting motif; ZF, zinc finger. **(B)** Top, molecular surface of p97 [N domain in white (Nn)/light gray (Nc), D1 in black, D2 in dark gray] with the SHP binding site indicated. Bottom, cartoon representation of the overall structure of the UFD1 SHP binding motif (colored in purple) bound to the p97 N domain (β-strands in dark gray and α-helices in light gray) (pdb entry 5B6C; Le et al., 2016). **(C)** Stick representations of the SHP binding motifs of UFD1 (left, pdb entry 5B6C, colored in purple; Le et al., 2016) and DER1 (right, pdb entry 5GLF, colored in purple; Lim et al., 2016b). The p97 N domain is shown as molecular surface (colored according to hydrophobicity). Key interactions are shown.

The SHP binding motif has been identified as an additional binding element in several UBX proteins and in UFD1, the latter typically forming a stable heterodimer with the UBXL protein NPL4 (Figure 4A). In contrast to the UBX/UBXL domains and the VIM/VBM binding motifs that bind into the hydrophobic cleft located in the N domain, the SHP binding motif targets an alternative binding site on the N domain. The SHP binding motif features two invariant glycine residues and a highly conserved aromatic residue with the consensus sequence h(x)_1−2_F/W(x)_0−1_GxGx_2_L (h, hydrophobic residue; x, any amino acid). The initial crystal structure of full-length p97 in complex with the SHP motif of UFD1 revealed that the motif adopts a mostly extended, yet slightly bent conformation, and binds at the periphery of the C-terminal α+β subdomain (Nc, aa 112–186) of the N domain, in direct vicinity of the ND1 linker (Hänzelmann and Schindelin, 2016a). Subsequently, a high-resolution structure of the N domain in complex with the UFD1-SHP using an N domain-SHP fusion protein (Le et al., 2016) (Figures 4B,C) as well as the N domain structure with the SHP of the DERLIN1 (DER1) rhomboid pseudoprotease (Lim et al., 2016b) (Figure 4C) were determined. The SHP motif forms a random coil interrupted by a small two amino acid long β-sheet, which associates with the central four-stranded β-sheet, thereby extending it to a five-stranded antiparallel β-sheet. In addition, an adjacent α-helix stabilizes the complex. The motif thus binds in a hydrophobic binding pocket and is stabilized between one of the β-strands and this α-helix, which together are arranged into a β-β-α super-secondary structure, a well-known binding mode mediating protein-protein interactions (Lim et al., 2016b). The two strictly conserved glycine residues (GxG) generate a sharp kink in the middle of the SHP motif, thereby enabling the bending of the motif upon binding to the N domain. The interaction mainly involves hydrophobic contacts with only a few electrostatic contacts being observed. Upon binding of the SHP motif flexible loop regions and secondary structural elements in the binding region are stabilized, including a loop region (^141^EAYRP^145^) found to be involved in UBX/UBXL and VIM/VBM interaction, thus suggesting that the rigidification of this region upon SHP binding may affect binding of other cofactors.

The interaction of the UFD1 SHP binding motif could be regulated by phosphorylation. Proteomic studies indicate that both serine residues (Ser229 and Ser231) located in the SGSG motif are phosphorylated, whereas no possible PTMs for the DER1 motif have been identified so far (Hornbeck et al., 2015).

### PUB and PUL domains

**Figure 5:**
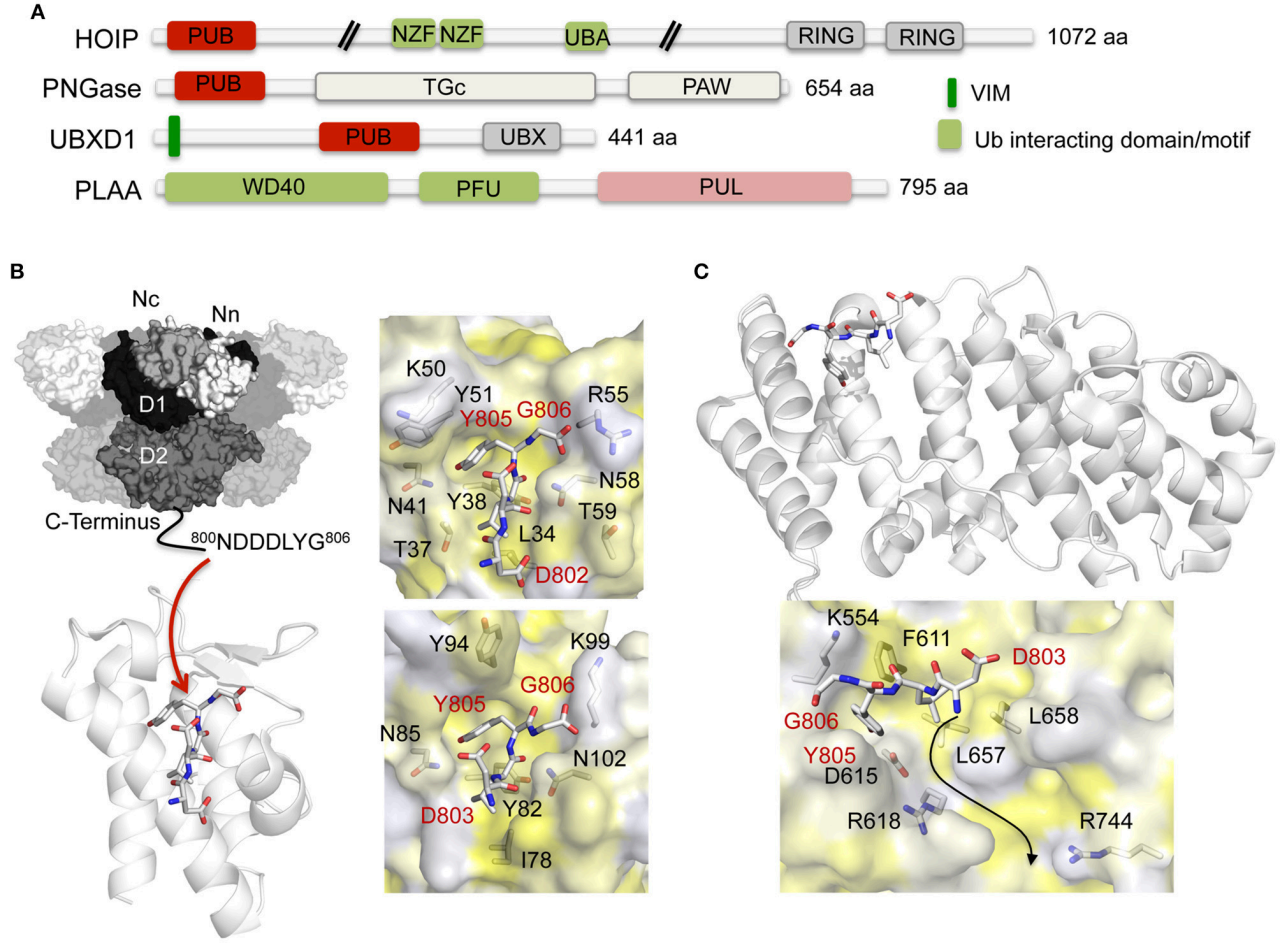
**Interaction of p97 with PUB and PUL domain-containing cofactors. (A)** Domain architecture of mammalian PUB and PUL domain-containing cofactors. NZF, NPL4 zinc finger; PAW, present in PNGases and other worm proteins; PFU, PLAA family ubiquitin binding domain; PUB, PNGase/UBA or UBX containing proteins; PUL, (PLAP, Ufd3, and Lub1p); RING, RING (Really Interesting New Gene) finger; TGc, transglutaminase like; UBA, ubiquitin-associated; UBX, ubiquitin regulatory X; WD40, WD40 β-propeller. **(B)** Left top, molecular surface of p97 [N domain in white (Nn)/light gray (Nc), D1 in black, D2 in dark gray] with the p97 C-terminal cofactor binding site indicated. Left bottom, overall structure of the p97 C-terminus (stick representation) bound to the PNGase PUB domain shown in cartoon representation (pdb entry 2HPL; Zhao et al., 2007) (adapted from Buchberger et al., 2015). Right, stick representation of the p97 C-terminus together with the molecular surface of the PNGase (top, pdb entry 2HPL, Zhao et al., 2007) and the HOIP (bottom, pdb entry 4P0A; Schaeffer et al., 2014) PUB domains (colored according to hydrophobicity). Key interactions are shown. **(C)** Top, overall structure of the p97 C-terminus (stick representation) bound to the PLAA PUL domain shown in cartoon representation (pdb entry 3EEB; Qiu et al., 2010) (modified from Buchberger et al., 2015). Bottom, stick representation of the p97 C-terminus together with the molecular surface of the PLAA PUL domain (colored according to hydrophobicity). The arrow indicates a possible location of the N-terminal extension of the p97 C-terminal residues. Key interactions are shown.

PUB and PUL domains, which are structurally unrelated, are currently the only known domains that interact with the extreme C-terminus of p97 (Figure 5A). Molecular insights into the p97-PUB/PUL domain interaction have been obtained by crystal structures of the PNGase (Zhao et al., 2007) and HOIP (HOIL-1-interacting protein; Schaeffer et al., 2014) PUB domains (Figure 5B) as well as of the PUL domain of PLAA (the ortholog of yeast Doa1/Ufd3, which is also known as phospholipase A2-activating protein or PLAP) (Qiu et al., 2010) (Figure 5C) bound to a peptide derived from the final residues of the p97 C-terminus, which is referred to as PUB interacting motif (PIM). The formation of the p97-PUB/PUL complex is mediated by hydrophobic and electrostatic interactions, with key interactions being contributed by the hydrophobic Leu804 as well as the aromatic side chain of the penultimate tyrosine residue (Tyr805) of p97, which inserts into a hydrophobic pocket on the PUB/PUL domain. Based on biochemical data (Zhao et al., 2009) it was proposed that the p97 C-terminus stretches into a neighboring positively charged ridge formed on the PUL surface (Figure 5C).

The direct interaction of the PUB/PUL-PIM interaction is regulated by PTMs. Phosphorylation of the strictly conserved penultimate tyrosine residue of p97 abolishes binding (Zhao et al., 2007; Li et al., 2008; Schaeffer et al., 2014), thus suggesting a conserved mechanism to control PIM interaction with their binding partners. In addition, PTMs of the respective PUB/PUL domain proteins could regulate the individual interactions. For example, proteomic studies (Hornbeck et al., 2015; Hendriks et al., 2017) revealed that the PUB domain of PNGase is ubiquitylated at Lys50, whereas in HOIP Lys99 is ubiquitylated. Furthermore, the PUB domain containing protein UBXD1 is phosphorylated at Tyr195 (corresponding to Tyr51 in PNGase), ubiquitylated at Lys180 and Lys202, which are replaced by threonines in PNGase (Thr37+59), as well as SUMOylated at Lys180 and Lys193 (the latter residue corresponding to Lys50 in PNGase). Finally, in the PUL domain of PLAA Lys554, which is located at a similar position to Arg55 of PNGase and Lys99 of HOIP, can be either acetylated, ubiquitylated or SUMOylated.

## Regulation of p97—cofactor assembly

**Figure 6:**
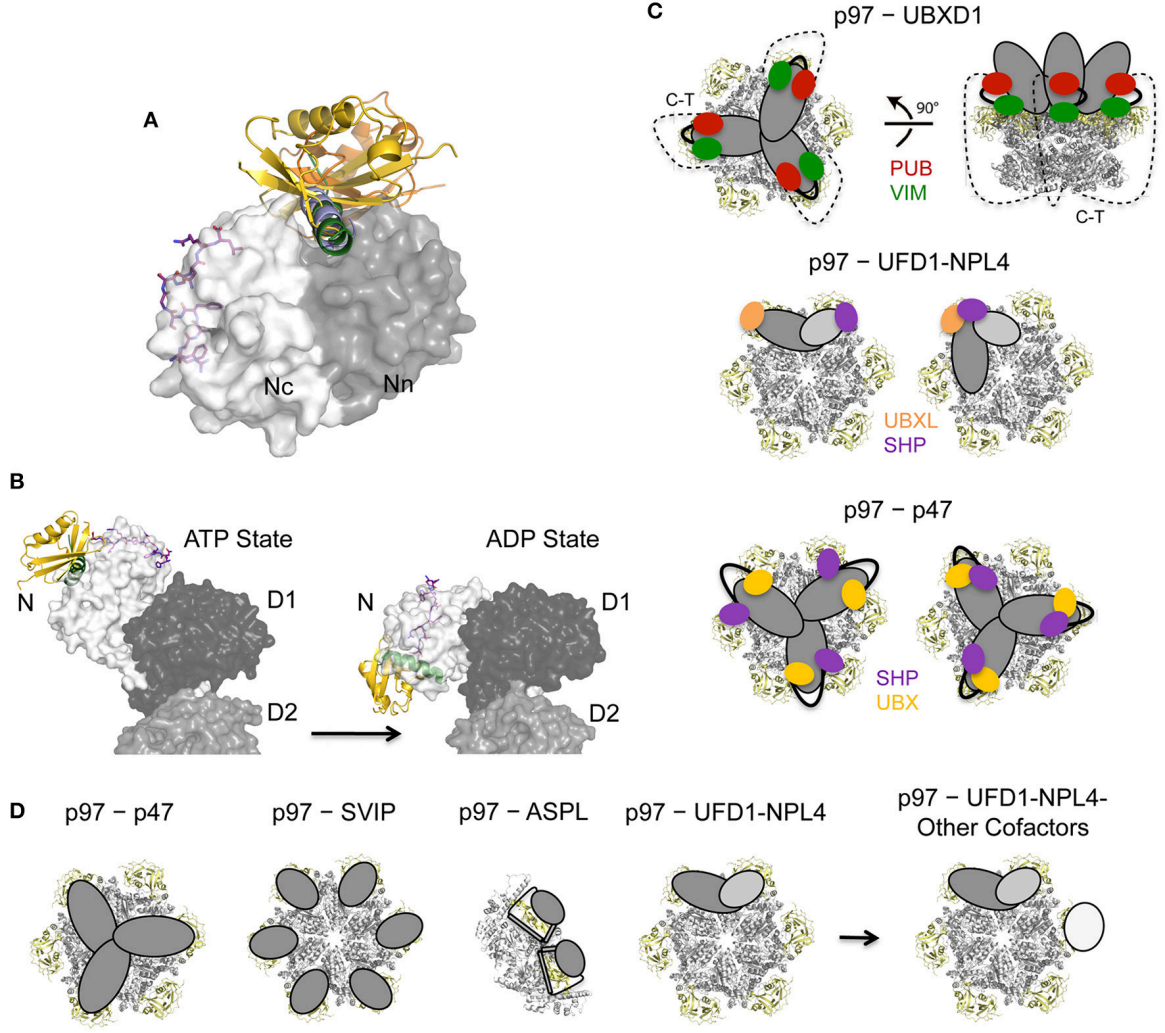
**Regulation of p97 cofactor binding to the N domain. (A)** Binding site competition. Superposition of p97 N domain cofactor complexes: SHP of UFD1 (pdb entry 5B6C, colored in purple; Le et al., 2016), VIM of gp78 (pdb entry 3TIW, colored in green; Hänzelmann and Schindelin, 2011), VBM of RHBDL4 (pdb entry 5EPP, colored in light blue; Lim et al., 2016b), FAF1-UBX (pdb entry 3QQ8, colored in gold; Hänzelmann et al., 2011) and NPL4-UBXL (pdb entry 4RV0, colored in olive; Hao et al., 2015). Cofactors are shown in cartoon or stick representation and p97 N as molecular surface (Nn in dark gray, Nc in light gray). **(B)** Conformational changes of p97 upon ATP binding/hydrolysis. Side view of the molecular surface of p97 in the ATP- and ADP-bound states (pdb entries 5FTN and 5FTK; Banerjee et al., 2016) together with a cartoon representation of the FAF1-UBX domain (pdb entry 3QQ8, colored in gold; Hänzelmann et al., 2011) and the gp78 VIM (pdb entry 3TIW, colored in green; Hänzelmann and Schindelin, 2011) as well as a stick representation of the UFD1 SHP binding motif (pdb entry 5B6C, colored in purple; Le et al., 2016). **(C)** Models for bipartite binding to p97. UFD1-NPL4 and p47 interact with either identical or different N domains (adapted with permission from Hänzelmann and Schindelin, 2016a) whereas UBXD1 simultaneously binds to the N domain and the unstructured C-terminus (C-T) (modified from Buchberger et al., 2015). p97 is shown in a cartoon representation with the N domains colored in yellow. **(D)** Models for different oligomeric assemblies and higher order complexes for different cofactors as indicated. p97 is shown in a cartoon representation with the N domains colored in yellow.

**Figure 7:**
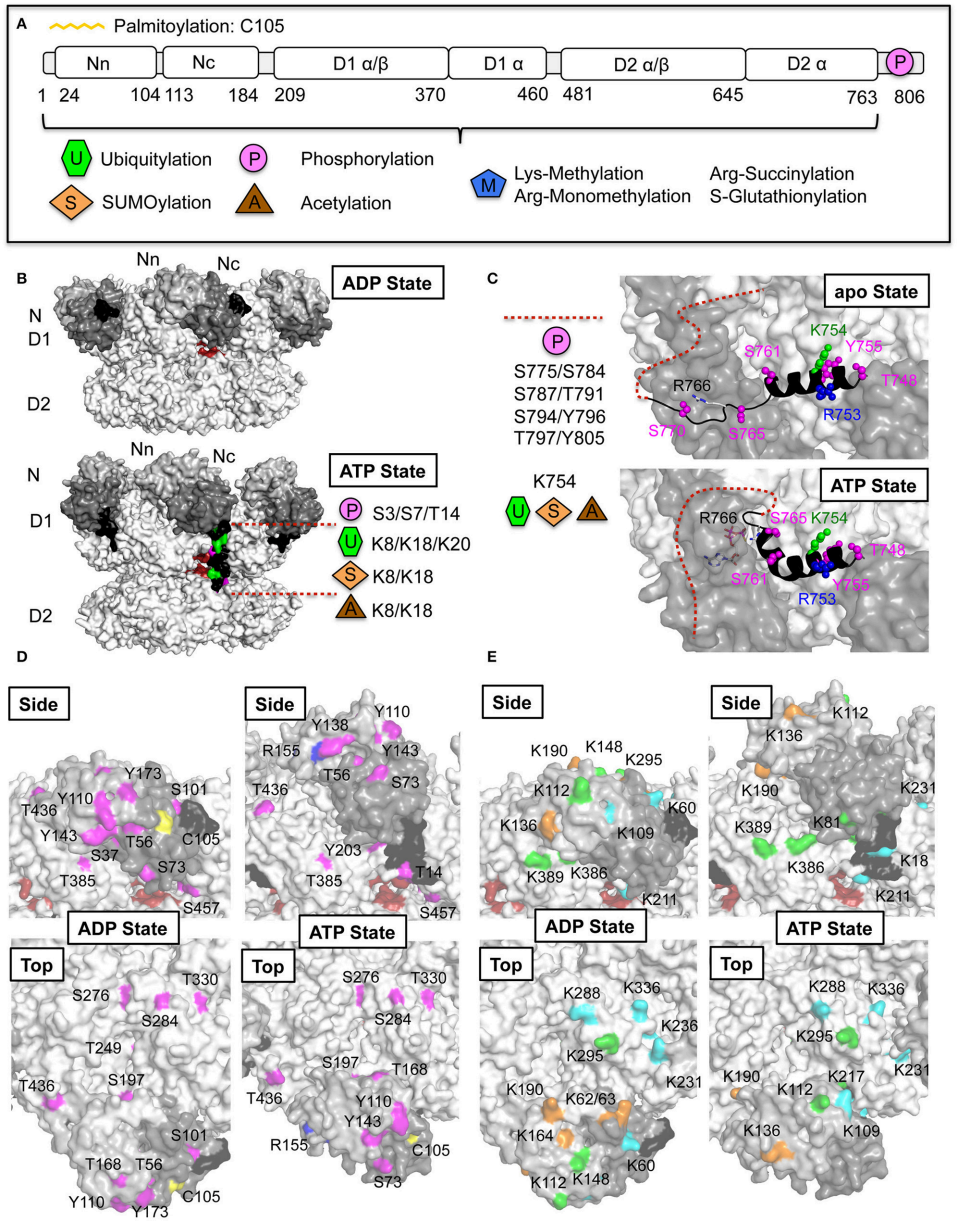
**p97 post-translational modifications (PTMs). (A)** Domain architecture of p97 together with identities of PTMs derived from the public database PhosphoSitePlus (Hornbeck et al., 2015) and published sources. **(B)** Nucleotide-induced conformational changes of the p97 N-terminal extension (residues 1–24, colored black). Molecular surface representation of p97 in the ADP and ATP states (pdb entries 5FTK and 5FTN; Banerjee et al., 2016). In one of the monomers of the ATP-bound structure the N-terminal extension is modeled according to Schuller et al. (2016). The opening at the D1D2 interface is shown in red. Identified PTMs on the extension are indicated. **(C)** Nucleotide-induced conformational changes of the p97 C-terminus. Molecular surface representation together with a cartoon representation of the C-terminus in the apo- (top) and ATP-bound state (bottom) (pdb entries 5C19 and 5C18; Hänzelmann and Schindelin, 2016b). The disordered C-terminal tail is indicated with dashed lines. Identified PTMs in the C-terminal helix α9 are shown and listed for the disordered region. **(D)** Phosphorylation sites identified in the ND1 part of p97 (N-terminal extension in black, N domain in dark gray, D1 in light gray). Identified phosphorylation sites (colored in magenta) are mapped onto the molecular surface of p97 in the ADP- and ATP-bound states (pdb entries 5FTK and 5FTN; Banerjee et al., 2016) and are shown in a side view and top view. Residues in the D1D2 interface are shown in red. In addition, palmitoylation of Cys105 (colored in yellow) and monomethylation of Arg155 (colored in blue) are indicated. **(E)** Ubiquitylation and SUMOylation sites identified on the ND1 part of p97. Identified ubiquitylation sites (colored in green) and SUMOylation sites (colored in orange) as well as sites, which carry both modifications (colored in cyan), are mapped onto the molecular surface of p97 as in **(D)**. p97 is colored as in **(D)**.

Since p97 participates in multiple cellular processes in different subcellular compartments, p97 needs to be specifically targeted to the respective pathway and its activity must be tightly controlled. On the one hand this is achieved through the diversity of its cofactors, however, cofactor assembly, in addition, is regulated by multiple mechanisms including binding site competition, bipartite binding, different binding stoichiometries, hierarchical binding, and conformational changes (Figure 6) (reviewed in Buchberger et al., 2015). Finally, *in cellulo* PTMs of p97 and its associated cofactors as well as crosstalk between PTMs introduce an additional level of complexity (Figure 7).

### Binding site competition

Overall, only three different interaction sites, the hydrophobic inter-subdomain cleft and the SHP binding site in the N domain together with the C-terminus of p97, have been identified so far and these must accommodate the 30 different cofactors, hence competition of diverse cofactors for the same site inevitably occurs. Specifically, although there is no sequence similarity between the VIM as well as VBM binding motifs and the UBX/UBXL domains, these cofactors all target the same general area, the hydrophobic interdomain cleft of the N domain, which explains the competitions between these cofactors *in vitro* (Figure 6A). Likewise, the PUB and PUL domain cofactors, which display no structural similarity to each other, bind in a highly conserved manner to the C-terminal tail. In cells, however, the situation is expected to be more complex. Depending on the presence of specific substrates and the subcellular localization certain cofactors may be more abundant or even exclusively present, thus alleviating the problem of cofactors competing for the same binding site.

### Conformational changes upon ATP binding and hydrolysis

ATP binding/hydrolysis is coupled to profound conformational changes in the location of the N domains which interconvert between a position in plane with the D1 ring (locked conformation or down conformation; ADP-bound state) to an orientation where they are located above the D1 ring (up conformation; ATP-bound state) (Banerjee et al., 2016; Schuller et al., 2016). Low-resolution cryo-EM studies of the p97−p47 (trimer), p97−FAF1 (trimer) and p97−UFD1-NPL4 complexes (Beuron et al., 2006; Bebeacua et al., 2012; Ewens et al., 2014) revealed that these interactions involve several N domains being present in the up conformation, hence suggesting that the up and down movement of the N domain during ATP-binding and hydrolysis could exert the necessary force to disassemble the macromolecular assemblies being targeted by p97 (Figure 6B). Furthermore, the association of cofactors with the N domain affects the ATPase activity of p97 in different ways including inhibitory as well as stimulatory effects (Trusch et al., 2015; Zhang et al., 2015), and it could be demonstrated that cofactors are recruited to the D1 domain in response to ATP binding (Chia et al., 2012).

### Bipartite p97-cofactor interactions

The two primary cofactors of p97, p47 and the heterodimeric UFD1-NPL4 complex as well as other cofactors like several UBX domain containing proteins, harbor more than one p97-binding module, which enables bipartite binding of these cofactors to the p97 N domain (Bruderer et al., 2004). In the case of p47 its UBX domain and SHP motif are involved, while the UFD1-NPL4 heterodimer employs the UBXL domain of NPL4 and the SHP motif of UFD1 to target p97. Low-resolution cryo-EM studies revealed that p47, where both binding domains/motifs reside on the same polypeptide, binds as a trimer where each subunit apparently interacts with two adjacent p97 monomers (Beuron et al., 2006). In case of the UFD1-NPL4 heterdimer it is still unclear how bipartite binding is accomplished. A model in which the NPL4-UBXL domain and the UFD1-SHP motif target adjacent N domains (Pye et al., 2007) lacks experimental support despite the existence of low-resolution EM structures depicting the p97−UFD1-NPL4 complex (Bebeacua et al., 2012). Recently it has been proposed that a bipartite binding could involve either a single or two adjacent N domains (Hänzelmann and Schindelin, 2016a) (Figure 6C).

UBXD1 also employs a bipartite binding mode in which its PUB domain interacts with the C-terminus of p97 and its VIM targets the N domain (Figure 6C) (Kern et al., 2009). Among p97 cofactors this interaction mode, which involves the two major binding sites of p97, namely the N domain and the C-terminus, being targeted by a single cofactor is unique, and is expected to restrict the conformational flexibility of p97. Isothermal titration calorimetry (ITC) studies demonstrated that UBXD1 binds as a trimer with both binding motifs contributing to this interaction (Hänzelmann and Schindelin, 2011).

However, it is currently unknown whether cofactors with two interaction sites remain stably associated via both binding modules at all times. Possibly, ATP hydrolysis and/or substrate binding may induce conformational changes leading to dissociation at one site with a concomitant increase in conformational flexibility of the complex during subsequent catalysis (Buchberger et al., 2015; Hänzelmann and Schindelin, 2016a). Hence a bipartite binding mode not only enhances the affinity of these cofactors but also imposes conformational restrictions in p97, which may modulate its catalytic properties.

### Oligomeric assembly

Interestingly, while p97 harbors six N domains most cofactors bind substoichiometrically (Figure 6D), probably due to steric hindrance of the large, multidomain cofactors. Currently, the only known cofactor that results in a 6:6 assembly is the small protein SVIP (Hänzelmann and Schindelin, 2011), which is an efficient competitor for N domain cofactors and has been suggested to be a negative regulator during ERAD (Ballar et al., 2007). Other cofactors like p47 and UBXD1 trimerize and, as discussed above, the UBX protein ASPL even disrupts the hexameric assembly and forms a heterotetrameric complex with p97. In contrast, only one UFD1-NPL4 heterodimer associates with two adjacent N domains within the p97 hexamer and additional cofactors could possibly interact with non-occupied N domains. Accordingly, it was demonstrated that UBXD7, UBXD8, FAF1 and SAKS1, which are all UBA-UBX domain containing proteins, coimmunoprecipitate with p97 bound to the UFD1-NPL4 heterodimer and endogenous ubiquitin conjugates (Alexandru et al., 2008), a finding which supports the existence of higher-order p97-cofactor1-cofactor2 complexes. Subsequently a hierarchical binding of the two UBA-UBX proteins FAF1 and UBXD7 was demonstrated (Hänzelmann et al., 2011; Lee et al., 2013). Specifically, for UFD1-NPL4 and FAF1, the resulting p97−UFD1-NPL4−FAF1 complex exhibited a stoichiometry of 6:1:1. Since no direct interaction between FAF1/UBXD7 and UFD1-NPL4 could be observed, it is conceivable that conformational changes are induced in p97 upon binding of UFD1-NPL4 which generate an asymmetry in p97 and as a consequence only one of the vacant p97 subunits has the ability to interact tightly with FAF1 or UBXD7 (Hänzelmann et al., 2011). In addition, a macromolecular complex composed of p97, UBXD1 and the two substrate-processing cofactors YOD1 and PLAA has been shown to be involved in the removal of ruptured lysosomes by autophagy, indicating that under certain conditions a distinct set of proteins interact with each other at least transiently in a substrate-dependent manner (Papadopoulos et al., 2017).

### Post-translational modifications (PTMs)

Recently it could be shown that most cofactors of p97 such as for example p47 form extremely dynamic complexes with p97 that undergo rapid dissociation and exchange in cell lysates, thus raising the question how p97-cofactor complexes can adequately perform their tasks *in vivo* (Xue et al., 2016). Mechanisms that modulate the lifespan and stabilization of p97-cofactor complexes like PTMs of p97 (Ewens et al., 2010) and its cofactors (Uchiyama et al., 2003; Almeida et al., 2015) or substrate recruitment by cofactors have been proposed.

PTMs like phosphorylation, lysine acetylation, and ubiquitylation function as molecular switches and may trigger or abolish the association of proteins with cofactors, lipids, DNA, and proteins. PTMs usually lead to structural changes such as exposing or masking active sites as well as interfaces for protein–protein interaction, thus regulating for example subcellular localization, stability, and activity in response to internal and external stimuli (reviewed in Beltrao et al., 2013; Ryšlavá et al., 2013; Venne et al., 2014). The abundance of PTMs is often controlled by so-called “writers” and “erasers,” which are enzymes capable of adding or removing the modifications, respectively. In the case of phosphorylation events these correspond to kinases and phosphatases while they involve E3 ligases and DUBs in the case of ubiquitylation. The functional consequences can also be exploited by proteins that specifically bind to these modifications, so called “reader” domains, like Scr-homology-2 (SH2) or WD40 domains, amongst many others, in the case of phosphorylation or specific ubiquitin-binding domains (UBD) in the case of ubiquitylation (reviewed in Seet et al., 2006; Beltrao et al., 2013).

Numerous high-throughput proteomic studies revealed that p97 is extensively targeted by PTMs like phosphorylation (66 identified sites), ubiquitylation (38 identified sites) and acetylation (24 identified sites) (PhosphoSitePlus, http://www.phosphosite.org, Hornbeck et al., 2015), however, the relevant enzymes and functional consequences of these modifications are poorly understood. Similar findings have been reported for other chaperones, which suggests the existence of a combinatorial code regulating the localization, activity, and substrate specificity for these biologically important proteins (Cloutier and Coulombe, 2013). Interestingly, in addition to modification sites that are only accessible in the functional hexameric form of p97, other sites were identified which are only partially accessible, completely buried or even located in the central channel. These latter sites would not be accessible for modifying enzymes unless p97 were to dissociate into a monomer, while partially buried sites could be accessible after conformational changes. Whether p97 can exist in a monomeric form and especially under which condition is not known. Currently the only known example is the described disruption of the hexamer through the interaction with the UBX protein ASPL resulting in the formation of a p97-ASPL heterotetramer (Arumughan et al., 2016). Beside numerous phosphorylation, ubiquitylation and acetylation events also SUMOylation, palmitoylation, methylation, succinylation, and S-glutathionylation were identified (Figure 7A). In the case of p97 PTMs may promote or prevent protein–protein interactions by obscuring existing binding sites, generating new interfaces, or triggering conformational changes, with the exact functional consequence dictated by the substrate and cellular context (Almeida et al., 2015).

Interestingly, in the small 24 amino acid long N-terminal extension (Figure 7B) as well as in the C-terminal tail with its preceding α-helix (α9) (Figure 7C) many PTMs have been identified, thus indicating an important function of these extensions in regulating the properties of p97. Recently it could be shown that the small N-terminal extension, which is highly flexible and disordered in the ADP-bound state undergoes a large conformational change upon ATP binding, when it becomes ordered, relocates itself beyond the cleft between the D1 and D2 domain and inserts into the D2 domain (Schuller et al., 2016) (Figure 7B). Furthermore, deletion of this extension reduces the ATPase activity to a similar degree as found in the absence of the N domain (unpublished data), suggesting an important function in the regulation of the ATPase activity. Furthermore, it could be demonstrated that the C-terminal α-helix undergoes a significant conformational change upon ATP binding (Figure 7C). This helix is kinked and inserts between two adjacent monomers into the ATP-binding pocket of the trans-monomer and an arginine (Arg766) directly coordinates the γ-phosphate of the ATP, thereby closing of the D2 nucleotide-binding pocket (Hänzelmann and Schindelin, 2016b). In the absence of nucleotide, the C-terminus is present in a different conformation and no longer inserts into the ATP-binding pocket. In addition to the N- and C-terminal extensions, the cofactor binding N domain and its associated D1 domain are also extensively targeted by PTMs (Figures 7D,E). In the following sections we will focus on PTMs that may have functional implications.

#### Phosphorylation

Protein phosphorylation, which typically targets serine, threonine, or tyrosine residues, is a reversible PTM controlled by kinases and phosphatases. Among the 66 currently known phosphorylation sites in p97 some have been studied in more detail. For example, the aforementioned phosphorylation of the C-terminal Tyr805 by c-Src kinase, which abolishes the interaction with PUB/PUL domain-containing cofactors (Zhao et al., 2007; Li et al., 2008; Schaeffer et al., 2014). The negatively charged phosphate group would undoubtedly introduce electrostatic and steric hindrance in the context of the tight binding pocket in the PUB/PUL domain.

Phosphorylation of the C-terminal tail residue Ser784 by DNA-PK (DNA-dependent protein kinase) was demonstrated to accumulate at sites of DNA double-strand breaks (DSBs) (Livingstone et al., 2005), where p97 interacts more tightly with chromatin. Furthermore, p97 can be phosphorylated on Ser457, Ser459, and Ser326 (buried) by ATM (ataxia telangiectasia-mutated) and ATR (ATM-Rad3-related), the two proximal checkpoint kinases, which regulate the DNA damage response (DDR) (Mu et al., 2007). Interestingly, Ser457 and Ser459 are both located next to the channel entrance at the D1D2 interface of the adjacent monomer (Figure 7D) and conformational changes can be expected upon phosphorylation.

Phosphorylation of Ser770 by SIK2 [salt inducible kinase 2 of the AMP–activated protein kinase (AMPK) family] stimulates the ATPase activity of p97 (Yang et al., 2013). Ser770 is located in the C-terminal tail, which undergoes significant conformational changes during ATP binding/hydrolysis (Figure 7C) (Hänzelmann and Schindelin, 2016b). Although SIK2 phosphorylates p97 through its N-terminal kinase domain, it interacts with p97 via its extremely glutamine-rich C-terminal region and plays a critical role in ERAD and ER homeostasis (Yang et al., 2013).

p97 is also a target of the serine/threonine kinase Akt (protein kinase B, PKB), which plays important roles in cell survival and phosphorylates p97 at Ser352 (buried), Ser746 (buried), and Ser748 (N-terminus of the C-terminal helix α9) (Klein et al., 2005; Vandermoere et al., 2006).

In the p97 N domain several phosphorylation sites identified in proteomic studies directly interfere with cofactor association (Figure 7D): (i) Thr37 and Ser56 are both located in the hydrophobic binding cleft; (ii) Tyr110 and Tyr143, which are both key residues for UBX/UBXL interaction; (iii) Thr168, which is located close to the SHP binding groove, and could influence binding of SHP-containing cofactors upon structural rearrangements. Since cofactors typically assemble on top of the D1 ring, phosphorylation at this position could influence this interaction by modulating cofactor affinity. Finally, phosphorylation sites in close proximity to the channel formed between the D1 and D2 domains were identified (see above).

#### Ubiquitylation

Covalent ubiquitin-protein conjugates are introduced in a series of three consecutive enzymatic steps (reviewed in Kerscher et al., 2006; Cappadocia and Lima, 2017). Initially a ubiquitin-activating enzyme (E1) activates ubiquitin in an ATP-dependent reaction and binds to it covalently. Subsequently, ubiquitin is transferred to a ubiquitin-conjugating enzyme (E2) in a trans-thioesterification reaction. Finally, ubiquitin is attached to one or more lysine residues in the target protein, in a reaction catalyzed by a ubiquitin ligase (E3). Modifications with ubiquitin are highly variable in length and linkage type (reviewed in Akutsu et al., 2016; Yau and Rape, 2016). Proteins can be modified at one or multiple lysine residues with either a single ubiquitin molecule (mono- and multi-monoubiquitylation, respectively) or ubiquitin polymers (polyubiquitylation). Modification of proteins with a single ubiquitin subunit typically alters intra- or inter-molecular interactions which in turn affect the localization, the activity of the modified protein or its ability to interact with partner proteins (Husnjak and Dikic, 2012). Ubiquitin contains seven lysine residues (Lys6, Lys11, Lys27, Lys29, Lys33, Lys48, and Lys63) among its 76 residues, which, together with its amino terminus, provide eight sites for attaching further ubiquitin moieties, resulting in homo- and heterotypic polymeric ubiquitin chains. Different linkage types lead to different conformations of the corresponding ubiquitin chains and hence in unique binding epitopes, which trigger specific downstream signaling events (reviewed in Liu and Walters, 2010; Akutsu et al., 2016; Yau and Rape, 2016). Ubiquitylation is a dynamic and reversible process. The action of the E1-E2-E3 cascade is counteracted by deubiquitylating enzymes (DUBs), which specifically remove ubiquitin from target proteins (reviewed in Husnjak and Dikic, 2012; Sahtoe and Sixma, 2015).

Several high-throughput proteomic analysis, which focused on ubiquitylation sites, identified a large number of p97 lysine residues that are ubiquitylated (Danielsen et al., 2011; Kim et al., 2011b; Wagner et al., 2011, 2012; Mertins et al., 2013; Elia et al., 2015; Wu et al., 2015). So far none of the sites have been analyzed in more detail, hence it is not known whether p97 is mono- or poly-ubiquitylated, and, should the latter be the case, the linkage types remain undefined. Also, it is not known which E3 ligases are involved in this process. However, p97 is known to associate with several E3 ligases in either a direct or indirect fashion. Direct interactions involve for example the HRD1 and gp78 E3 ligases (Ballar et al., 2006; Morreale et al., 2009), which are both involved in ERAD, or HOIP (Schaeffer et al., 2014), the E3 ligase of the LUBAC complex (Linear Ubiquitin Chain Assembly Complex), which is involved in the formation of linear ubiquitin chains. Indirectly, p97 interacts with SCF E3 ligases containing cullins that bind for example to UBXD7, which in turn binds via its UBX domain to p97 (Alexandru et al., 2008). In addition, the DUBS ATAXIN-3, VCIP135 and YOD1 are known p97 interaction partners (Uchiyama et al., 2002; Boeddrich et al., 2006; Ernst et al., 2009). The physiological function of p97 ubiquitylation remains undefined, but has been speculated to modulate its affinity for cofactors or substrates. Alternatively, the modifications could shift the equilibrium between the different conformational states of p97 or, in the extreme case, even induce additional conformational states. Monoubiquitylation of p97 could of course be a signal to recruit the protein to specific cellular compartments.

Ubiquitylation sites in p97 have been identified in the N, D1, and D2 domain, but not in the flexible C-terminal extension (Figure 7E). Taking into account the size of ubiquitin of 8.5 kDa in relation to the p97 N domain (21 kDa), one can conclude, that pretty much irrespective of the site where the modification is introduced, ubiquitylation of the N domain would interfere with cofactor interactions. Ubiquitylation of the N-terminal extension (Lys8/18/20) could prevent insertion of the N-terminus into the D2 domain and could block the entrance to the putative substrate-binding channel (Figure 7B). Furthermore, ubiquitylation on top of the D1 ring would directly interfere with cofactor interactions, which typically assemble above the D1 ring (Figure 7E). Ubiquitylated p97 could then be recognized by proteins that contain at least one UBD like for example the helical binding domains UBA, UIM, and CUE, zinc fingers like NZF and UBZ as well as other proteins harboring Jab1/MPN, PFU, and WD40 domains (Husnjak and Dikic, 2012). All these domains were identified in several p97 cofactors.

#### SUMOylation

SUMOylation is a ubiquitin-related reversible conjugation pathway in which members of the SUMO family (SUMO1 or the highly related SUMO2/3) are attached to lysine residues of target proteins via an isopeptide bond through the sequential action of E1, E2, and E3 enzymes (reviewed in Cappadocia and Lima, 2017). The participating enzymes can discriminate between the SUMO paralogs at both the conjugation and deconjugation levels (Citro and Chiocca, 2013). Although the two ubiquitin-like modifiers SUMO and ubiquitin are structurally related and feature a β-grasp fold, they have different molecular properties. Besides the presence of three different SUMO isoforms, all of them feature an additional N-terminal extension and a different surface charge. These properties are responsible for different activating, conjugating and deconjugating enzymes and distinct cellular functions (reviewed in Praefcke et al., 2012; van der Veen and Ploegh, 2012). Furthermore, in contrast to ubiquitin chains, which can be linked through all seven lysine residues, Lys11 in the N-terminal extension is the major SUMO acceptor site in SUMO2/3, whereas SUMO1, which is lacking a SUMOylation consensus site, is mainly involved in monoSUMOylation (Matic et al., 2008). In contrast to ubiquitylation, SUMOylation preferentially targets disordered and flexible protein regions (Hendriks et al., 2017). SUMOylation typically controls the dynamics of protein assemblies through binding of SUMO conjugates to SUMO recognition modules termed SUMO interaction motifs (SIMs), whereas ubiquitin interacting proteins typically bind via their UBD to the hydrophobic patch around Ile44 of ubiquitin. A number of SUMOylated protein targets feature the consensus motif “Ψ-K-x-E/D,” (Ψ, hydrophobic residue; x, any amino acid). Recent proteomic studies demonstrated that a considerable fraction of total SUMOylation events involve non-consensus sites (Blomster et al., 2010), especially under stress conditions when SUMOylation loses stringency and can act like ubiquitylation. Whereas ubiquitylation, acetylation and phosphorylation events occur throughout the cell, SUMOylation takes place predominantly in the nucleus, more specifically in chromatin and nuclear bodies (Hendriks and Vertegaal, 2016). The degree of SUMOylation is dynamically regulated by various forms of stress, thereby linking SUMOylation to the regulation of cellular homeostasis (Liebelt and Vertegaal, 2016), where it plays ubiquitin-dependent and independent roles. A tightly regulated balance in both time and space exists between ubiquitin and SUMO, in which the same lysine within a protein is targeted and this determines the function, localization, or stability of the modified protein.

By direct comparison of the endogenous SUMO1- and SUMO2/3-modified proteome in mammalian cells p97 was found to be preferentially conjugated to SUMO1 (81%; Becker et al., 2013). Accordingly, it could be shown that the p97 N domain is modified with SUMO1 in a dynamic process involving several non-consensus sites, suggesting that different lysine residues could compensate for each other (Wang et al., 2016). Site directed mutagenesis studies indicate that Lys60/62/63, Lys136 as well as Lys164 (Figure 7E) are the most important sites involved in SUMOylation under stress conditions. SUMOylation of p97 under conditions of oxidative and ER stress leads to the distribution of p97 to stress granules and into the nucleus, and promotes assembly of the p97 hexamer. In contrast, pathogenic N domain mutations identified in MSP and FALS, which feature an uncoordinated conformational change of the N domain due to a disturbed communication between the N and D1 domains (reviewed in Tang and Xia, 2016), lead to reduced SUMOylation and weakened p97 hexamer formation upon stress (Wang et al., 2016). Cryo-EM studies of a pathogenic mutant revealed highly flexible N domains, which are situated more on top of the D1 ring, reflecting the ATP-bound state observed in the wild type (Niwa et al., 2012). Since in the ATP-bound state Lys60/62/63 and Lys164 are protected, this would explain why in pathogenic states p97 SUMOylation is reduced. However, SUMOylation of wild type p97 at these positions would also affect N domain movement, ATPase activity and the interaction with cofactors. Defects in the SUMOylation of p97 also trigger altered cofactor binding and attenuated ER-associated protein degradation (Wang et al., 2016). A recent comprehensive SUMO2-specific proteomic study of mammalian cells under standard growth conditions and stress conditions identified 17 additional SUMOylation sites distributed over all domains of p97 (Hendriks et al., 2017). Most of the SUMO targeted lysine residues are also found to be ubiquitylated indicating a crosstalk between SUMOylation and ubiquitylation (see below). However, the SUMO1 targeted N domain residues Lys62, Lys63, and Lys 136 as well as the SUMO2 targeted residue Lys190, which is located in the ND1 linker, are exclusively modified by SUMO.

In addition, the p97 cofactor UFD1, which together with NPL4 is frequently found to be involved in chromatin associated processes, harbors seven SUMOylation sites in its predicted disordered C-terminal region, which is located downstream of the p97 SHP binding motif (Hendriks et al., 2017).

There are several indications that p97 operates at the intersection of the ubiquitylation and SUMOylation pathways, two major signaling events which target chromatin. Consequently, it was proposed that p97, through its ability to associate with cofactors displaying affinities for ubiquitin and SUMO, links these two pathways to either trigger protein degradation or elicit other regulatory events (Bergink et al., 2013; Franz et al., 2016; Nie and Boddy, 2016). The SUMO targeted ubiquitin ligase (STUbl) family of proteins (Sriramachandran and Dohmen, 2014; Nie and Boddy, 2016) integrates ubiquitin and SUMO modifications into a hybrid signal. The resulting mixed SUMO-ubiquitin chains can be recognized by the UFD1-NPL4 complex, which contains both ubiquitin (NZF domain of NPL4, UT3 domain of UFD1) and SUMO (UFD1 C-terminus) interacting motifs.

SUMO conjugation and de-conjugation processes might play a role in p97 functions during DNA repair to regulate the recruitment and release of the participating proteins. A similar function has been shown for the AAA ATPase MDN1, which acts as a SUMO-targeted regulator in mammalian pre-ribosome remodeling (Raman et al., 2016).

#### Palmitoylation (S-Acylation)

Palmitoylation, myristoylation, and prenylation are the most frequently identified covalent lipid modifications (reviewed in Hentschel et al., 2016). Of these three lipid modifications, only palmitoylation is reversible, thus allowing for a more dynamic regulation of protein function with respect to trafficking, localization, stability, aggregation, and interaction with effectors (reviewed in Cho and Park, 2016). Lipidation increases the hydrophobicity of proteins, which promotes the association of the modified proteins with the plasma membrane and other membranes such as those of the ER, mitochondria, Golgi, and endosomes. Palmitoylation, which is catalyzed by palmitoylacyltransferases (PATs), also known as DHHC enzymes and is reversed by palmitoyl protein thioesterases, is the covalent attachment of the 16 carbon fatty acid palmitate to the side chain of specific cysteine residues of target proteins via a thioester bond. Dependent on the target protein palmitoylation functions in a large variety of cellular processes including subcellular trafficking as well as signal transduction and aberrant palmitoylation has been associated with Alzheimer's disease, Huntington's disease and other neurodegenerative disorder (Cho and Park, 2016).

Palmitoylation of p97 at Cys105 located in the N domain has been reported (Fang et al., 2016) (Figure 7D). Palmitoylation of p97 could be important for participation of p97 in a variety of cellular processes involved in the regulation of membrane fusion and vesicular trafficking, however, the significance of p97 palmitoylation has not been addressed so far.

#### ε-Acetylation

Besides phosphorylation and ubiquitylation, protein acetylation is probably the most frequent and important PTM involved in cell signaling, gene expression, stress responses, apoptosis, membrane trafficking as well as cellular metabolism and plays a major role in the regulation of nuclear proteins, in particular histones (reviewed in Drazic et al., 2016). Acetylation is catalyzed by lysine (K) acetyltransferases (KATs), which transfer the acetyl group from acetyl-coenzyme A (Ac-CoA) to the ε-amino group of lysine residues. This process is reversible and tightly regulated. Malfunctions of the acetylation machinery have been implicated in cardiovascular and neurodegenerative diseases as well as cancer (Drazic et al., 2016).

The importance of p97 in a large variety of chromatin associated processes like transcription, replication, and DNA repair suggests that p97 is regulated through acetylation. For example, intracellular accumulations of abnormal proteins such as expanded polyglutamines in neuronal cells induces p97 phosphorylation at Ser612 and Thr613 as well as acetylation of Lys614, which allows p97 to translocate into the nucleus (Koike et al., 2010). Following translocation general transcription is suppressed via deacetylation of core histones, resulting in cell atrophy and inhibition of *de novo* protein synthesis, which decreases the accumulation of misfolded proteins, thus allowing the cell to remove them by chaperone-mediated refolding, proteasomal degradation, and autophagy (Koike et al., 2010). The location of the three sequential residues Ser612, Thr613, and Lys614 in the D1D2 interface close to the entry points to the central channel suggests that conformational changes occur in this region.

High-throughput proteomics (Hornbeck et al., 2015) identified a total of 24 putative p97 acetylation sites, which, interestingly, all overlap with ubiquitylation and SUMOylation sites (Hornbeck et al., 2015; Hendriks et al., 2017) indicating competition between the enzymes catalyzing the different PTMs (see below). This includes acetylation of the N-terminal extension residues Lys8 and Lys18 (Figure 7B), several lysines of the N domain and the aforementioned Lys614 as well as Lys754 in helix α9 preceding the C-terminal tail (Figure 7C).

#### Lysine and arginine N-Methylation

Methylation is a PTM, which influences protein-protein interactions, activity, and turnover of proteins as well as cellular localization (reviewed in Biggar and Li, 2015). The ε-amino group of lysine may be modified with up to three methyl groups by lysine-specific methyltransferases (KMTs) and the side chain of arginine may be mono- or di-methylated by arginine methyltransferases (PRMTs; reviewed in Biggar and Li, 2015). S-Adenosylmethionine (SAM; also known as AdoMet) serves as the methyl donor in both reactions. The addition of methyl groups to lysine and arginine residues may negatively alter hydrogen bond-mediated interactions or, alternatively, facilitate stacking with aromatic residues as the methylated residues become more hydrophobic, thus increasing the structural diversity of proteins and modulating their cellular functions. Similar to protein phosphorylation, protein methylation plays important roles in signaling pathways involved in cell growth and differentiation and has been associated with several diseases including cancer (Biggar and Li, 2015).

It could be demonstrated that METTL21D (VCP lysine methyltransferase, VCP-KMT) can tri-methylate p97 on Lys315 (Kernstock et al., 2012). Interestingly, Lys315 is located buried inside the p97 channel close to the constriction in the D1 domain formed by the His-gate and thus not accessible to a methyltransferase in the hexameric state. However, it could be shown that methylation was stimulated by ASPL (Cloutier et al., 2013), indicating that this site becomes available after disruption of the p97 hexamer. An additional lysine mono-methylation site has been identified on Lys231 (Hornbeck et al., 2015), which is located on top of the D1 ring, which has also been found to be ubiquitylated/SUMOylated (Hornbeck et al., 2015; Hendriks et al., 2017). In addition, a recent proteome-wide analysis of arginine mono-methylation sites (Larsen et al., 2016) identified five arginines in p97, which are all functionally important: (i) Arg155, which is the most frequently mutated residue found in MSP (Figure 7D); (ii) Arg586 and Arg599, which are both located in the D2 pore-loop 2; (iii) Arg708 located in a regulatory loop region on the outside of the D2 domain (Hänzelmann and Schindelin, 2016b); (iv) Arg753 located in the C-terminal helix (Figure 7C).

#### S-glutathionylation

Reactive oxygen/nitrogen species (ROS/RNS) have been found to act as important physiological modulators of intracellular signaling pathways, but are also causative of aging, cancer, neurodegenerative disorders, and cardiovascular diseases (reviewed in Finkel, 2011; Chung et al., 2013). Covalent modifications of selected cysteine residues present in redox-sensitive proteins mediate, at least in part, the specific effects of ROS/RNS. Oxidative PTMs (Ox-PTM) of cysteine residues represent an important mechanism that regulates protein structure and, ultimately, function. Ox-PTMs including S-nitrosylation (also called S-nitrosation, SNO), sulfhydration (SSH), S-glutathionylation (SSG), disulfide bond formation (RS-SR), and sulfenylation (SOH) are stimulated by diffusible small molecules and constitute reversible modifications. In addition, the irreversible formation of sulfinic (SO_2_H) and sulfonic acids (SO_3_H) on cysteine residues are induced.

It could be shown that under conditions of oxidative stress p97 is S-glutathionylated at Cys522 (Noguchi et al., 2005). Cys522 is present in the ATP-binding pocket of the D2 domain and its modification negatively regulates the ATPase activity of p97, thus leading to ER stress. Addition of glutathione to Cys522 would induce steric hindrance interfering with ATP binding on the D2 domain. Cys522 modification leads to an accumulation of ubiquitylated proteins and ER stress, followed by apoptosis, which are phenotypes found in several neurodegenerative disorders (Noguchi et al., 2005).

#### Crosstalk between various protein translational modifications

Proteins are often regulated via a combination of different PTMs, possibly acting as a molecular barcode or PTM code (Beltrao et al., 2013; Venne et al., 2014). These modifications may trigger specific effectors to either initiate or inhibit downstream events, which either induce or retain a signal only when the complementary incoming signal occurs simultaneously both in time and space. The interplay between different PTMs, referred to as crosstalk (reviewed in Beltrao et al., 2013; Venne et al., 2014), can be either positive or negative (Hunter, 2007). Phosphorylation-dependent ubiquitylation (Koepp et al., 2001) and SUMOylation (Hietakangas et al., 2006) represent examples of positive crosstalk where the initial PTM serves as active trigger for the subsequent addition or removal of a second PTM, or as a recognition site for other proteins. Short crosstalk motifs like phosphodegrons involved in ubiquitin-mediated protein degradation and, in general, motifs in which a phosphorylation site is simultaneously present with another PTM, a second phosphorylation site, or SUMOylation/acetylation sites in the context of a five amino acid stretch are known (Ye et al., 2004; Yao et al., 2011). Negative crosstalk may result from the direct competition of two PTMs for the same amino acid or from indirect effects due to one specific PTM masking the recognition site of a second PTM (Hunter, 2007). For example, direct competition exists between SUMOylation, ubiquitylation, phosphorylation, and acetylation with ubiquitylation/SUMOylation and SUMOylation/acetylation being mutually exclusive, while SUMOylation/phosphorylation can be agonistic or antagonistic depending on the substrate in question (Escobar-Ramirez et al., 2015). Furthermore, the combination of different PTMs on a protein generates a highly regulated interface which may be recognized by specific effector proteins resulting in the controlled initiation of downstream signaling events and facilitating the interactions with diverse binding partners (Sims and Reinberg, 2008), thus explaining, for example, how p97 can participate in such a large variety of different cellular functions.

In the case of p97 one would envision a negative crosstalk between acetylation, lysine methylation, ubiquitylation and SUMOylation since all these PTMs compete with each other for the same lysine residues. Also, the high number of PTMs identified in p97 indicates that a combinatory code is at play that regulates its activity, function, substrate specificity, and localization (Cloutier and Coulombe, 2013). The identification of functionally relevant sites and their dynamic regulations requires quantitative mass-spectrometry approaches that can measure changes in the abundance of PTMs under different conditions (Beltrao et al., 2013). For example a recent global profiling study of ubiquitylation, phosphorylation and acetylation in the DNA damage response identified for p97 located in the nucleus several ubiquitylation and acetylation sites, although no phosphorylation sites were found under the same experimental conditions (Elia et al., 2015).

## Models for substrate unfolding and disassembly activity

**Figure 8:**
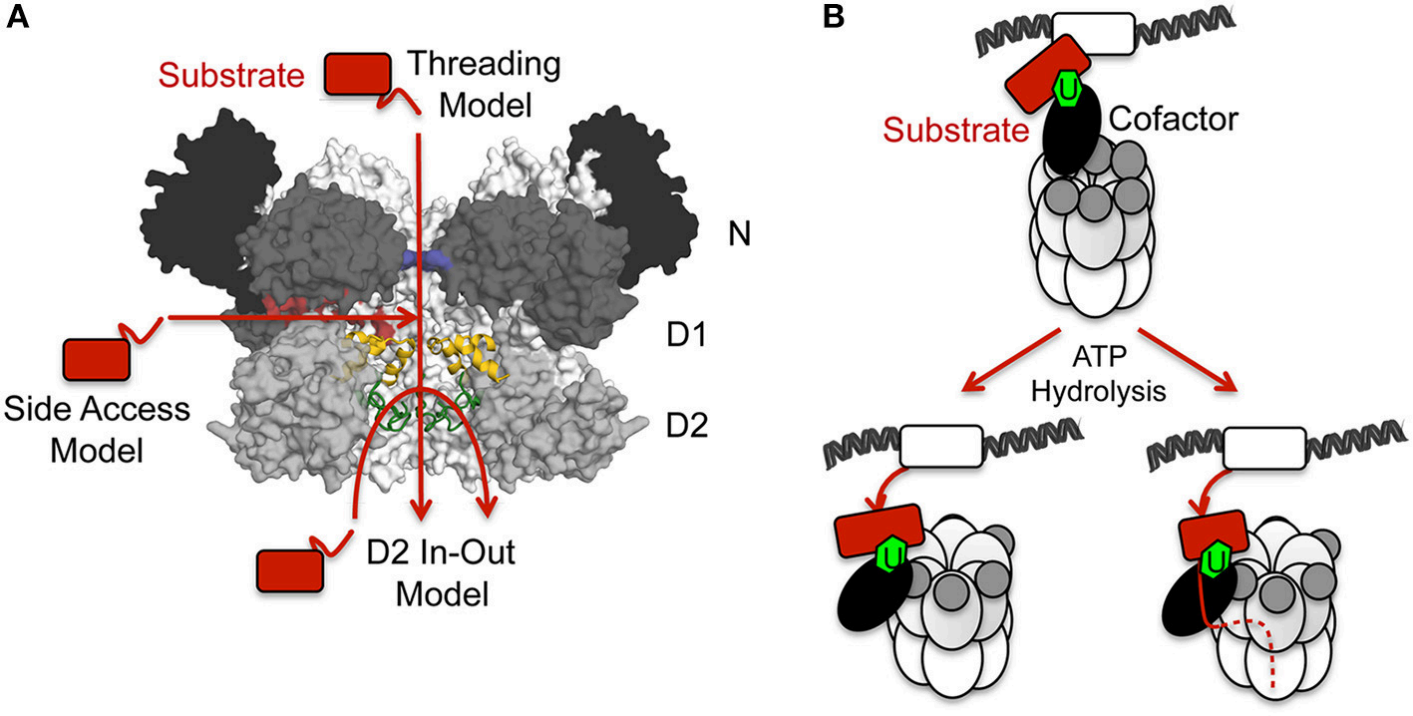
**Models of p97 disassembly activity. (A)** Proposed p97 translocation pathways. Molecular surface of the p97 central cavity (side view) in the ATP bound state (pdb entry 5FTN; Banerjee et al., 2016) with two monomers in black/dark/medium (N/D1/D2) gray and two monomers in light gray. For clarity, the two monomers in the front are not shown. The restriction in the D1 domain (His-gate) is shown in blue, the two D2 pore loops are colored in yellow and forest, respectively and the opening at the D1D2 interface is shown in red. Proposed translocation pathways according to the different models (threading, side access and D2 in-out) are indicated with red arrows. **(B)** Models for the disassembly function of p97 (translocation-independent model). In the ATP-bound state the N domains are in the up-position and interact via bound cofactors for example with a ubiquitylated substrate being part of a chromatin-associated protein complex. Upon ATP-hydrolysis the down-movement of the N domain would exert a force on the bound substrate, which would disassemble it from the protein complex (left). Depending on the substrate, alternatively the down-movement of the N domain would bring the cofactor bound substrate to the D1D2 interface and unfolded regions of the substrate could enter through this path into the p97 D2 pore with its putative substrate binding loops, which could provide an additional pulling force (right, hybrid model).

Conformational changes triggered by ATP binding and hydrolysis generate mechanical forces which are responsible for the activity of p97 in the unfolding and disassembly of macromolecular complexes. In the case of p97 the underlying mechanism(s) is (are) still unknown and different models have been proposed (Figure 8): (i) The threading model in which substrates are threaded through the central pore of p97; (ii) The D2 in-out model where substrates insert and leave the D2 pore from the D1-distal direction; (iii) The side access model according to which substrates enter the protein chamber through the opening between the D1 and D2 interface; (iv) The translocation-independent or disassembly model which implicates movements of the N domain rather than a direct participation of the D2 pore in the mechanism. These hypotheses will be discussed in more detail in the following paragraphs.

- The threading model (Figure 8A): In this model the substrate initially inserts into the D1 ring from where it is completely translocated along the axial channel and is released on the D2 site for subsequent degradation by the 26S proteasome (Ye et al., 2003). There is clear evidence that other AAA+ unfolding machines, including the bacterial ClpX and ClpA enzymes as well as the proteasomal Rpt1–6 motors (reviewed in Bar-Nun and Glickman, 2012; Bittner et al., 2016) utilize this mechanism. Molecular dynamics simulations of p97 also suggest this mechanism (Tonddast-Navaei and Stan, 2013), however, high-resolution cryo-EM structures of p97 in different nucleotide states suggest that the axial pore in the D1 ring remains too narrow to accommodate a peptide during ATP hydrolysis due to the constriction imposed by the His-gate formed by His317 (Banerjee et al., 2016; Schuller et al., 2016). Furthermore, the D1 ring does not have pore loops as found in the D2 domain and probably cannot bind substrates. Furthermore, only His317 is critical for catalysis, while other D1 pore lining residues were found to not be important (DeLaBarre et al., 2006). Therefore, a substrate translocation through the entire channels seems unlikely.
- The D2 in-out model (Figure 8A): Here substrates insert and leave the D2 pore from the D1-distal direction while being processed in the D2 pore (DeLaBarre et al., 2006). The D2 pore contains the typical substrate binding loops found in related enzymes, which, depending on the nucleotide status, are either in a fixed or dynamically released conformation (Davies et al., 2008; Hänzelmann and Schindelin, 2016b). The smaller pore loops contain a conserved Φ-X-Gly (aromatic-hydrophobic-Gly) tripeptide motif which has been suggested to play a conserved role during substrate translocation by AAA+ unfoldases (reviewed in Sauer and Baker, 2011; Olivares et al., 2016). However, since all major substrate-recruiting cofactors bind to the N domain residing at the opposite end of p97 relative to the D2 domain, it is hard to imagine how a substrate can enter the D2 pore from the bottom.
- The side access model (Figure 8A): Substrates possibly enter the D2 pore without passing through the D1 pore by entering through a side portal located in between the D1 and D2 rings. A recent normal mode analysis of p97 (Na and Song, 2016) shows that upon ATP binding the D1 and D2 domains move slightly apart from each other, thus forming an additional channel at the D1D2 interface, which leads into the D2 pore. The up and down movements of the N domain may serve to translocate substrates to the interface between the D1 and D2 domains where they can enter into the hexamer. The limited chamber volume suggests that a substrate may be expelled at the wide end of the D2 ring while at the same time it is pulled in at the D1D2 interface. The importance of the D1D2 interface channel is supported by studies from the Bruenger lab (DeLaBarre et al., 2006). Cross-linking experiments of the substrate synaptotagmin and p97 identified Lys565, which is located adjacent to the pore loop-1 residues Trp551/Phe552 and is located within the proposed D1D2 interface channel. Whether the proposed channel is of sufficient size to accommodate a polypeptide is currently unknown. However, it is imaginable that substrate-binding induces larger conformational changes, possibly aided by PTMs in the vicinity of the entry points at the D1D2 interface. Ubiquitylation/SUMOylation of Lys211 (Figure 7E) and phosphorylation of Ser457 (Figure 7D), Ser459 as well as Ser462 have been found (Mu et al., 2007; Hornbeck et al., 2015; Hendriks et al., 2017).
- The translocation-independent or disassembly model (Figure 8B). At least for substrates that are recycled and not degraded by the proteasome a translocation-independent model seems likely (Buchberger, 2013). This would be related to the function of the AAA+ ATPase N-ethylmaleimide sensitive factor (NSF), which, together with its cofactor SNAP, disassembles the highly stable SNARE complexes forming after each membrane fusion event (reviewed in Zhao and Brunger, 2016). p97 undergoes large conformational changes during ATP binding and hydrolysis including the up and down movement of its N domains. Upon ATP binding to the D2 and D1 domains longitudinal motions of the D1 pore are coupled to the up-movement of the N domains, and the N domains, via bound cofactors, can interact with substrates. ATP hydrolysis in the D1 domain, which causes the N domain to return to the down position, recruits the substrate to the D1D2 interface as in the side access model. Nucleotide-dependent conformational changes in p97 would exert a force on the bound substrate, which would remove it from a macromolecular assembly.

Most likely, the mechanism by which p97 unfolds/disassembles target proteins depends on the fate of the substrate (Barthelme and Sauer, 2016), specifically whether it is recycled or partially/fully unfolded for degradation. Furthermore, in the presence of a ubiquitylated substrate and/or PTMs larger conformational changes in the D1 pore and the D1D2 interface region may occur. Nevertheless, an unfoldase activity of p97 as well as an involvement of the p97 central pore in substrate translocation is still hypothetical at this point since the critical residues located in the D2 pore loops play important roles in regulating the ATPase activity of p97 (DeLaBarre et al., 2006; Hänzelmann and Schindelin, 2016b). Hence one cannot interfere from mutagenesis data whether these side chains contribute to ATP hydrolysis or translocation. As mentioned above, an ISS couples the conformation of the pore to the nucleotide state of the same subunit, which is then transmitted to the adjacent subunit and, in this way, coordinates ATP hydrolysis in trans (Hänzelmann and Schindelin, 2016b). A hybrid model takes into account the importance of the p97 pore loops in substrate remodeling, yet it does not require substrates to completely translocate through the axial channel (Barthelme and Sauer, 2016). With the aid of its pore loops p97 could exert a force on a peptide segment of the substrate, leading to its deformation and dissociation from the complex without unfolding the substrate or translocating it through its axial channel (Figure 8B).

## Concluding remarks

A common function of p97 is its ATP-dependent extraction or disassembly of ubiquitylated substrates from chromatin, membranes, and protein complexes in many diverse cellular functions that maintain cellular homeostasis, contribute to genomic stability and govern important signaling pathways (Figure 1B). The key questions regarding the biological functions of p97 are how it participates in so many dissimilar cellular processes in different cellular compartments, in particular, how is p97 targeted to specific cellular pathways and recognizes its substrates and decides on their fates whether they are destined for proteasomal degradation or recycled. Therefore, independent regulatory mechanisms are necessary to control the physiological functions of p97. It is well-established that a large variety of substrate recruiting and substrate processing cofactors provide specificity toward the cellular processes p97 is involved in. Although cofactor assembly is regulated by binding site competition, bipartite binding, conformational changes upon ATP binding/hydrolysis and the formation of specialized subcomplexes composed of several cofactors, the situation in cells, however, is expected to be more complex and PTMs of p97 and its associated cofactors as well as a crosstalk between PTMs introduce an additional level of complexity. A total of about 170 PTMs like phosphorylation, ubiquitylation, acetylation, SUMOylation, palmitoylation, and methylation have been currently identified in p97, indicating that a combination of different PTMs affects the activity, localization, and substrate specificity of p97 in different cellular pathways. In the high-throughput proteomics era the list of p97 cofactors, associated substrates and PTMs is expected to grow and additional cellular functions may emerge. Major challenges for the future will be (i) to establish the correlations between biological functions and the many PTMs reported to exist, (ii) to identify proteins/domains that can specifically recognize them and (iii) to investigate the interplay of p97-cofactor interactions with PTMs. Understanding these aspects will be crucial for elucidating the physiological and patho-physiological functions of p97.

## Author contributions

PH primarily wrote this review with input from HS. Both authors approved it for publication.

### Conflict of interest statement

The authors declare that the research was conducted in the absence of any commercial or financial relationships that could be construed as a potential conflict of interest.
